# Defects boost graphitization for highly conductive graphene films

**DOI:** 10.1093/nsr/nwad147

**Published:** 2023-05-19

**Authors:** Qing Zhang, Qinwei Wei, Kun Huang, Zhibo Liu, Wei Ma, Zehui Zhang, Yanfeng Zhang, Hui-Ming Cheng, Wencai Ren

**Affiliations:** Shenyang National Laboratory for Materials Science, Institute of Metal Research, Chinese Academy of Sciences, Shenyang 110016, China; School of Materials Science and Engineering, University of Science and Technology of China, Shenyang 110016, China; Shenyang National Laboratory for Materials Science, Institute of Metal Research, Chinese Academy of Sciences, Shenyang 110016, China; School of Materials Science and Engineering, University of Science and Technology of China, Shenyang 110016, China; Shenyang National Laboratory for Materials Science, Institute of Metal Research, Chinese Academy of Sciences, Shenyang 110016, China; School of Materials Science and Engineering, University of Science and Technology of China, Shenyang 110016, China; Shenyang National Laboratory for Materials Science, Institute of Metal Research, Chinese Academy of Sciences, Shenyang 110016, China; School of Materials Science and Engineering, University of Science and Technology of China, Shenyang 110016, China; Shenyang National Laboratory for Materials Science, Institute of Metal Research, Chinese Academy of Sciences, Shenyang 110016, China; School of Materials Science and Engineering, University of Science and Technology of China, Shenyang 110016, China; School of Materials Science and Engineering, Peking University, Beijing 100871, China; School of Materials Science and Engineering, Peking University, Beijing 100871, China; Shenyang National Laboratory for Materials Science, Institute of Metal Research, Chinese Academy of Sciences, Shenyang 110016, China; School of Materials Science and Engineering, University of Science and Technology of China, Shenyang 110016, China; Shenzhen Institute of Advanced Technology, Chinese Academy of Sciences, Shenzhen 518055, China; Shenyang National Laboratory for Materials Science, Institute of Metal Research, Chinese Academy of Sciences, Shenyang 110016, China; School of Materials Science and Engineering, University of Science and Technology of China, Shenyang 110016, China

**Keywords:** graphene film, defects, graphitization, electrical and thermal conductivity, thermal management and EMI shielding

## Abstract

Fabricating highly crystalline macroscopic films with extraordinary electrical and thermal conductivities from graphene sheets is essential for applications in electronics, telecommunications and thermal management. High-temperature graphitization is the only method known to date for the crystallization of all types of carbon materials, where defects are gradually removed with increasing temperature. However, when using graphene materials as precursors, including graphene oxide, reduced graphene oxide and pristine graphene, even lengthy graphitization at 3000°C can only produce graphene films with small grain sizes and abundant structural disorders, which limit their conductivities. Here, we show that high-temperature defects substantially accelerate the grain growth and ordering of graphene films during graphitization, enabling ideal AB stacking as well as a 100-fold, 64-fold and 28-fold improvement in grain size, electrical conductivity and thermal conductivity, respectively, between 2000°C and 3000°C. This process is realized by nitrogen doping, which retards the lattice restoration of defective graphene, retaining abundant defects such as vacancies, dislocations and grain boundaries in graphene films at a high temperature. With this approach, a highly ordered crystalline graphene film similar to highly oriented pyrolytic graphite is fabricated, with electrical and thermal conductivities (∼2.0 × 10^4^ S cm^−1^; ∼1.7 × 10^3^ W m^−1^ K^−1^) that are improved by about 6- and 2-fold, respectively, compared to those of the graphene films fabricated by graphene oxide. Such graphene film also exhibits a superhigh electromagnetic interference shielding effectiveness of ∼90 dB at a thickness of 10 μm, outperforming all the synthetic materials of comparable thickness including MXene films. This work not only paves the way for the technological application of highly conductive graphene films but also provides a general strategy to efficiently improve the synthesis and properties of other carbon materials such as graphene fibers, carbon nanotube fibers, carbon fibers, polymer-derived graphite and highly oriented pyrolytic graphite.

## Teaser

The high-temperature defects induced by nitrogen doping significantly accelerate the grain growth and ordering of graphene films during graphitization, enabling highly-oriented-pyrolytic-graphite-like highly conductive films.

## INTRODUCTION

Graphene is well known for its extraordinary in-plane electron and phonon transport on a honeycomb lattice [1]. Transferring these remarkable properties, on a molecular level, into macroscopic laminate films is essential for many applications such as electromagnetic interference (EMI) shielding and thermal management [2–7], which are increasingly in demand with the explosive development of highly integrated and high-speed fifth-generation (5G) wireless mobile devices [8,9]. However, electron and phonon transports in graphene laminates are limited by lattice defects (e.g. vacancies, heteroatoms and grain boundaries (GBs)) and structural disorders (e.g. turbostratic stacking, voids, gaps and crumples) [6,7,10,11]. Thus, it is desirable to synthesize a highly ordered crystalline graphene film with large grain size, so that its overall behavior approaches that of single-crystal graphite. To this end, high-temperature graphitization is required for repairing defects and promoting graphite grain growth and ordering [6,7,12–15], and it is the only method known to date for the crystallization of all types of carbon materials, including graphene fibers [16], carbon nanotube (CNT) fibers [17], carbon fibers [18], polymer-derived graphite [19] and highly oriented pyrolytic graphite (HOPG) [20].

Graphene oxide (GO) [6,21–23] is the most common precursor for fabricating graphene films because of its excellent processability induced by oxygen functional groups, however such groups are decomposed to gaseous H_2_O, CO and CO_2_ during the thermal annealing process. The resulting structure expansion prohibits grain growth and ordering of the graphene film during graphitization. As alternative precursors, graphene materials with less functional groups, such as reduced GO (rGO) [12], a mixture of GO and rGO [13], pristine graphene sheets [14] and even large-area highly crystalline graphene films grown by chemical vapor deposition (CVD) [15], have been used to efficiently avoid film expansion. For all these precursors, during the thermal annealing process, the in-plane defects are gradually restored as the temperature is raised and almost disappear at ∼2000°C [24]. Nevertheless, the products suffer from small grain sizes and abundant structural disorders, even after graphitization at 3000°C for up to a few hours, due to high energy barriers for the migration and rearrangement of carbon atoms in the absence of lattice defects [25], which are key to the crystallization and ordering of graphitic materials.

Here, we show that nitrogen doping can retard the restoration of defects in graphene films below 2000°C and therefore retain abundant vacancies, dislocations and GBs at the beginning of graphitization. Such high-temperature defects substantially accelerate the grain growth and ordering of graphene films during graphitization, producing a HOPG-like highly crystalline film with electrical and thermal conductivities approaching the limit of single-crystal graphite within 10 min of graphitization at 3000°C. From 2000°C to 3000°C, the grain size of graphene films increases 100-fold, along with well-ordered AB-stacked layers being formed, leading to a 64- and 28-fold increase in electrical and thermal conductivities, respectively. The electrical and thermal conductivities (∼2.0 × 10^4^ S cm^−1^; ∼1.7 × 10^3^ W m^−1^ K^−1^) of the resulting graphene films are approximately six and two times better, respectively, compared to those fabricated by the commonly used GO precursor. Such a graphene film also exhibits a superhigh EMI-shielding effectiveness (SE) of ∼90 dB at a thickness of 10 μm, outperforming all synthetic materials of comparable thickness, including MXene films.

## RESULTS AND DISCUSSION

**Figure 1.**
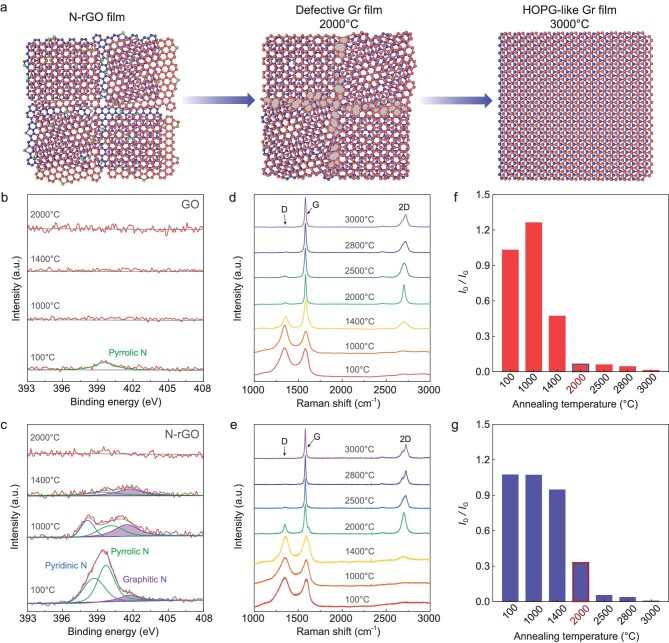
Creating high-temperature defects in graphene films at 2000°C by nitrogen doping. (a) Schematic of the structure evolution from N-rGO films to HOPG-like graphene (Gr) films with increasing annealing temperature. The green balls represent the doped nitrogen atoms and the gray fillings represent the GBs in a graphene lattice. (b and c) N 1s XPS spectra of the as-synthesized GO (b) and N-rGO (c) films, and after annealing at 1000°C, 1400°C and 2000°C. (d and e) Raman spectra evolution with increasing annealing temperature from 100°C to 3000°C for GO (d) and N-rGO (e) films. (f and g) The corresponding *I*_D_/*I*_G_ of GO (f) and N-rGO (g) films after thermal annealing at different temperatures.

Figure 1a shows a schematic diagram of the structure evolution from nitrogen doped-rGO (N-rGO) laminates to HOPG-like graphene films with annealing temperature. Nitrogen doping was realized by treating GO sheets under a hydrothermal condition with the addition of ammonia [26]. After treatment, the thickness and lateral size of the sheets remain unchanged (Supplementary Fig. 1), while the nitrogen content increases from ∼1.5 wt% to ∼10.3 wt% along with a reduction, with C/O atomic ratio rising from 1.4 to 5.4 (Fig. 1b and c, Supplementary Fig. 2 and Table 1). The trace nitrogen in GO exists in the form of pyrrolic N (Fig. 1b), while the resulting N-rGO sheets contain pyridinic N, pyrrolic N and graphitic N (Fig. 1c). The N-doping enables N-rGO sheets to have good dispersion in water even when most oxygen was removed (Supplementary Fig. 3). The graphene films were fabricated by filtrating N-rGO dispersions into laminates first, and then heating to 3000°C for 10 min graphitization followed by natural cooling and cold pressing (Supplementary Fig. 3).

We first studied the composition and structure evolution of N-rGO and GO films up to 2000°C. X-ray photoelectron spectroscopy (XPS) and elemental analysis were used to monitor the evolution of carbon, oxygen and nitrogen species in the films during annealing (Fig. 1b and c, Supplementary Fig. 4 and Supplementary Table 1). Notably, both the abundant oxygen and trace pyrrolic N in GO are nearly completely removed after annealing at 1000°C. For N-rGO, as the heat treatment temperature increases, oxygen species are nearly completely removed as well, but there is still a large amount of N species left after 1000°C annealing. In particular, compared to the oxygen-containing groups, N species such as pyridinic and pyrrolic N, bonding to two carbon atoms at the edges or defects of graphene, and graphitic N bonding to three carbon atoms in the graphene lattice, are more stable and can survive temperatures over 1400°C. A previous study shows that substitutional N species can still be observed in GO that is highly reduced by hydrozine after 1800°C annealing [27]. However, nearly no nitrogen species could be detected in the N-rGO films at 2000°C (Fig. 1c, Supplementary Table 1).

It is well accepted that GO is composed of isolated and intact sp^2^ graphene domains surrounded by oxidized sp^3^ regions that are decorated by epoxide and hydroxyl groups on their basal plane, and its edges are decorated with carbonxyles and carbonyls [27]. Raman spectra show that the as-synthesized GO and N-rGO films are both highly disordered with numerous defects (Fig. 1d and e). During thermal annealing, the oxygen-containing groups in GO films are decomposed to gaseous H_2_O, CO and CO_2_ to generate a great number of vacancies in the carbon lattice, corresponding to the increased intensity ratio of D peak to G peak (*I*_D_/*I*_G_) at 1000°C. However, the *I*_D_/*I*_G_ significantly declines from 1000°C to 2000°C, indicating that the in-plane defects are well-repaired below 2000°C (Fig. 1d and f). This is consistent with previous observations, which show that the recovery of a graphene lattice can be attained between 1800°C and 1950°C [27]. In sharp contrast to GO films, the *I*_D_/*I*_G_ of N-rGO films changes slightly until 1400°C and still has a high value at 2000°C (Fig. 1e and g), which is ∼5 times larger than that of GO films after 2000°C annealing (Fig. 1f). This result indicates that the presence of stable N species significantly retards the restoration of the graphene lattice to retain abundant defects at 2000°C.

We then studied the structure evolution of N-rGO and GO films during annealing from 2000°C to 3000°C. The Raman 2D peak, as the second harmonic of D peak, is usually used for measuring the degree of stacking order. A symmetrical 2D peak appears at 2700 cm^−1^ (2D_T_) for disordered turbostratic stacking, while AB stacking leads to an asymmetrical 2D peak composed of two subpeaks at ∼2680 cm^−1^ of (2D_1_) and ∼2720 cm^−1^ (2D_2_) [28,29]. As shown in Fig. 1d and e, compared to the films obtained below 2000°C, the significant rise of the 2D peak at 2000°C indicates the initial formation of ordered structures in graphene films, consistent with the high-resolution transmission electron microscopy (HRTEM) observations (Supplementary Figs 5 and 6), but only with turbostratic stacking in terms of the symmetric 2D peak profile. Further increasing the temperature leads to the restoration of lattice defects and transformation from turbostratic stacking to AB stacking, evidenced by the suppressed D peak and asymmetric 2D peak (Fig. 1d and e). These results suggest that the graphite grain growth and ordering in graphene films (i.e. graphitization) mainly occur above 2000°C, similar to the graphitization of other carbon materials [30]. Surprisingly, after 3000°C graphitization, N-rGO-derived graphene films show much higher crystallinity than GO-derived ones, although the former has many more defects and disorders than the latter at 2000°C (Fig. 1d–g), indicating that the structural features of graphene films at 2000°C play a key role in graphitization behavior.

**Figure 2.**
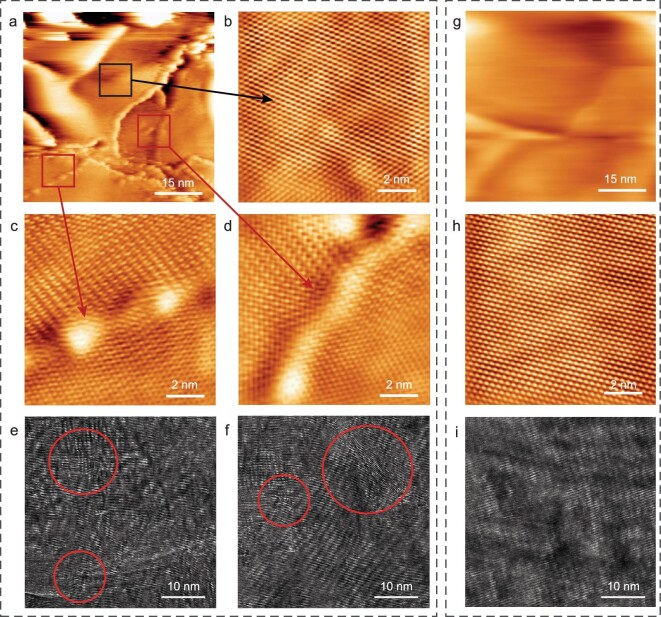
Structure characterizations of N-rGO and GO films obtained at 2000°C. (a–f) Nanometer-scale STM image (a), the corresponding atomically resolved STM images at different regions (b–d), and cross-sectional HRTEM images (e and f) of N-rGO film obtained at 2000°C, showing edges (a), pristine region (b), GBs (c and d) and dislocations, stacking faults and rotational mismatch (e and f). The defects are indicated by red arrows (c and d) and circles (e and f). (g–i) Nanometer-scale STM image (g), the corresponding atomically resolved STM image (h), and cross-sectional HRTEM image (i) of GO film obtained at 2000°C, showing a well-restored and ordered structure.

Therefore, we further characterized the in-plane and through-plane microstructures of GO and N-rGO films after 2000°C annealing by using scanning tunneling microscopy (STM) and HRTEM, respectively. For the N-rGO films, nanometer-STM images show many edges (Fig. 2a), which are possibly created by mechanical cleavage to expose a fresh surface [31]. Importantly, in addition to the pristine graphene regions (Fig. 2b), the atomically resolved STM images show clear GBs even in a small region (Fig. 2c and d), where the neighboring grains show different lattice orientations [31,32]. Such GBs are easy to break due to having lower strength than the pristine regions [33], which is the main reason for the generation of abundant edges. Cross-sectional HRTEM images show a microcrystalline structure with abundant defects, including dislocations, GBs, stacking faults and rotational mismatch (Fig. 2e and f). In addition, considering the removal of stable N species above 1800°C, the generation of vacancies in the N-rGO films could not be excluded. In contrast, for the GO films at 2000°C, both STM and HRTEM images show a well-restored and ordered structure (Fig. 2g–i).

**Figure 3.**
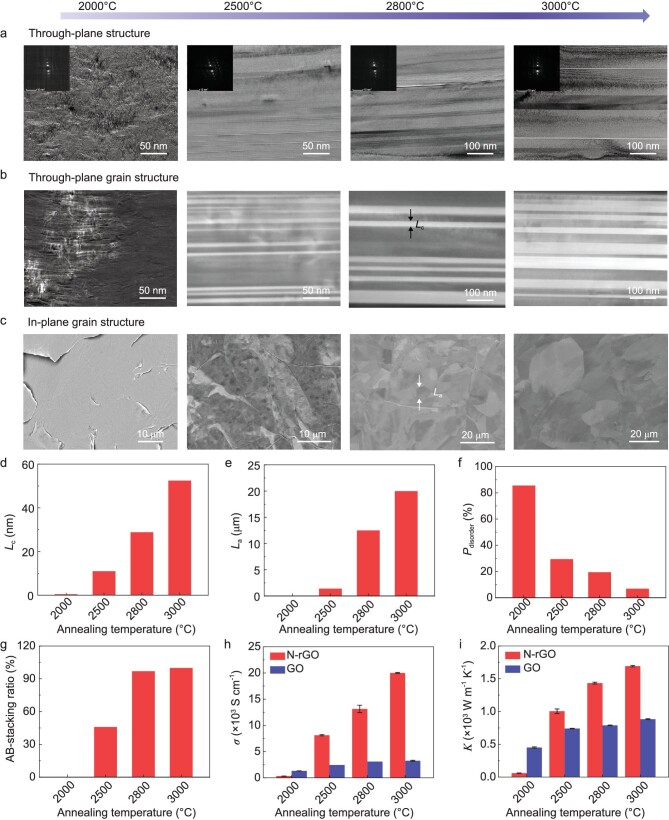
Structure and property evolution of N-rGO film during graphitization. (a) Cross-sectional BF-TEM images of N-rGO films with the corresponding selected area electron diffraction patterns (insets). (b) Cross-sectional ADF-STEM images. (c) SEM-ECC images of the film surface. The films from left to right were obtained after annealing at 2000°C, 2500°C, 2800°C and 3000°C, respectively. (d–g) Evolutions of *L*_c_ (d), *L*_a_ (e), *P*_disorder_ (f), the fraction of AB stacking (g), electrical conductivity (h) and thermal conductivity (i) of N-rGO films with annealing temperature. To show the role of high-temperature defects, the evolutions of electrical and thermal conductivities of GO films are also presented in (h) and (i), respectively.

Bright-field TEM (BF-TEM), annular dark-field scanning TEM (ADF-STEM), HRTEM and scanning electron microscopy-electron channel contrast (SEM-ECC) imaging were then used to record the evolution of the graphite grain in N-rGO films during the graphitization process above 2000°C (Fig. 3a–c). In SEM-ECC images, the in-plane graphite grains with different orientations show different brightnesses because of the different backscattered electron densities [19], which allows the determination of in-plane grain size (*L*_a_). X-ray diffraction (XRD) has been previously used to evaluate the through-plane grain size (*L*_c_) of graphene films and other carbon materials based on the Scherrer equation [12,13,22,34], but many instrumental and non-instrumental factors can influence the determination of *L*_c_, such as experimental resolution, grain shape, grain size distribution, degree of stacking order, microstrain and other defects [35–37]. Thus, ADF-STEM imaging was used here to characterize the grain size along through-plane directions in graphene films. An ADF-STEM detector could collect the Bragg diffracted electrons, of which the number approaching the detector is determined by the crystal orientation [38]. As a result, the single crystals with different orientations show different brightnesses in ADF-STEM images, which allows the exact determination of *L*_c_.

As shown above, N-rGO films exhibit the morphology of fragmentized crystallites with abundant defects at 2000°C (Figs 2a–f, 3a and b). Surprisingly, the grain growth and ordering develop rapidly from 2000°C to 2500°C. Cross-sectional BF-TEM and ADF-STEM images clearly show that graphene film at 2500°C is composed of parallel graphite grain bands with different orientations recognized by brightness difference (Fig. 3a and b). Simultaneously, the in-plane graphite grains are observed by using SEM-ECC imaging, but they show a small grain size of ∼1.4 μm and weak contrast (Fig. 3c). With further increasing the annealing temperature, coalescence occurs between adjacent grain bands, leading to substantial improvements in *L*_c_ (Fig. 3b). Meanwhile, the in-plane grain contour becomes much clearer and *L*_a_ expands greatly (Fig. 3c). HRTEM images clearly demonstrate the improvement of crystallinity and ordering of graphite structure in N-rGO films during the above graphitization process (Supplementary Fig. 6).

We further quantified the evolution of *L*_c_ and *L*_a_ as annealing temperature increases during graphitization, based on cross-sectional ADF-STEM and SEM-ECC images (Fig. 3d and e). At the same time, we evaluated the degree of ordering of graphene films quantitatively according to the *d*-spacing extracted from XRD data (Fig. 3f and Supplementary Table 2), performed Lorentzian fitting on the 2D peaks to identify the fraction of AB stacking based on the intensity ratio of 2D_2_/(2D_2_ + 2D_T_) (Fig. 3g, Supplementary Table 2), and measured their in-plane electrical and thermal conductivities using the four-probe method and laser flash technique, respectively (Fig. 3h and i, Supplementary Table 3). Notably, when the temperature is increased from 2000°C to 2500°C, *L*_c_ substantially increases from ∼0.5 to 11 nm, the proportion of disoriented layers (*P*_disorder_) decreases from ∼85.1% to 29.4%, the AB-stacking ratio increases to 45.8%, and the electrical and thermal conductivities increase ∼26 and 17 times, respectively. More importantly, *L*_c_ and *L*_a_ further enlarge ∼5- and 14-fold, respectively, and *P*_disorder_ further decreases by ∼4 times with ideal AB stacking being formed, as the temperature increases from 2500°C to 3000°C. Accordingly, the electrical and thermal conductivities are further increased 2.5- and 1.7-fold, respectively. In total, the grain size, electrical conductivity and thermal conductivity are increased 100-, 64- and 28-fold, respectively, during graphitization from 2000°C to 3000°C.

For comparison, we studied the structure and property evolution of GO films from 2000°C to 3000°C. Figure 3h and i, Supplementary Figs 5 and 7, and Tables 2 and 3 show that their grain size, ordering and properties develop slightly as the annealing temperature increases. At 2000°C, the in-plane defects in GO films are well repaired due to the low energy barrier. This makes them superior to N-rGO films at 2000°C in both electrical and thermal conductivities. However, the electrical and thermal conductivities of GO-derived graphene films are only increased ∼2.5- and 2-fold, respectively, from 2000°C to 3000°C. Such improvements are ∼26 and 14 times worse than those of N-rGO-derived graphene films. In particular, the conductivity increase is less than 35% from 2500°C to 3000°C. It is surprising to note that the electrical and thermal conductivities of N-rGO-derived graphene films at 2500°C are over two, and one, times higher than those of GO-derived films at 3000°C. Such property evolution is consistent with the slow growth and ordering of graphite grain in GO films. From 2000°C to 3000°C, *L*_c_ is increased only ∼9-fold in total, along with a moderate decrease in *P*_disorder_ from 84.0% to 28.7% and a subtle improvement in crystallinity (Supplementary Figs 5 and 7, and Supplementary Table 2).

As reported previously [13], the removal of oxygen in N-rGO can reduce film expansion (Supplementary Figs 8 and 9), which is beneficial to improving the ordering and properties of the graphene film. To reveal the dominant factor boosting graphitization, we also synthesized rGO sheets by hydrothermal treatment without ammonia and N-rGO with the same C/O atomic ratio (3.6) and 9.3 wt% nitrogen by changing the temperature and time of hydrothermal treatment in the presence of ammonia (Supplementary Fig. 10 and Table 1), which avoided the influence of oxygen on the formation of graphene films. After 2000°C annealing, 9.3 wt% N-rGO shows a much higher *I*_D_/*I*_G_ than that of rGO (Supplementary Fig. 11), indicating that many defects are retained by N-doping. These N-rGO-derived graphene films show *L*_c_, *L*_a_, and electrical and thermal conductivities of ∼28.6 nm, 6.5 μm, 1.2 × 10^4^ S cm^−1^ and 1.4 × 10^3^ W m^−1^ K^−1^ (Supplementary Fig. 12), respectively, which are much larger than those of rGO-derived graphene films (∼16.5 nm, 3.2 μm, 9400 S cm^−1^ and 1000 W m^−1^ K^−1^) (Supplementary Fig. 13 and Table 3). All the above comparisons demonstrate the dominant role of N-doping, which induced high-temperature defects in boosting the grain growth and ordering of graphene films.

It is well accepted that AB stacking is the most thermodynamically stable structure of graphite, while stacking faults and rotational mismatch can induce significant interlayer strain energy, which results in the crystals having a high-energy metastable state [39]. Previous work shows that the migration and annihilation of high-energy GBs in graphene can decrease the total energy of the system and are thermodynamically favored [40]. Importantly, they can result in the restoration of the graphene lattice. Moreover, the interlayer stacking faults and rotational disorders in multilayered 2D polycrystals can be healed by GB sliding when thermally activated, which is driven by the enhancement in interlayer vdW binding energy when the high-energy turbostratic stacking transfers into the low-energy AB stacking [39]. Taking into account the abundant GBs present in the N-rGO films at 2000°C, this explains the significantly improved grain growth and ordering of N-rGO films during graphitization. In addition, other defects such as vacancies and dislocations are all high energy active structure features and easy to move upon thermal annealing [27,41], and therefore may also facilitate graphitization. According to molecular dynamic simulations and first-principles calculations, the energy barrier is ∼3.6–3.9 eV when a single vacancy migrates from one graphene layer to a neighboring defective graphene layer, and it increases to as high as 7.3 eV when migrating to a perfect graphene layer [25]. Thus, it is easier for interlayer vacancy migration and the resulting carbon atom rearrangement to occur in defective graphene laminates than in well-restored graphene laminates. In contrast to N-rGO, when using GO as precursor, the defects are mostly restored before 2000°C, which limits the migration and rearrangement of carbon atoms, resulting in small improvements in grain size, ordering and properties during graphitization (also see Supplementary Figs 5 and 7, and Tables 2 and 3). This also explains the small grain size and mixed stacking order in the graphene films synthesized from less defective even highly crystalline graphene precursors [12–15].

**Figure 4.**
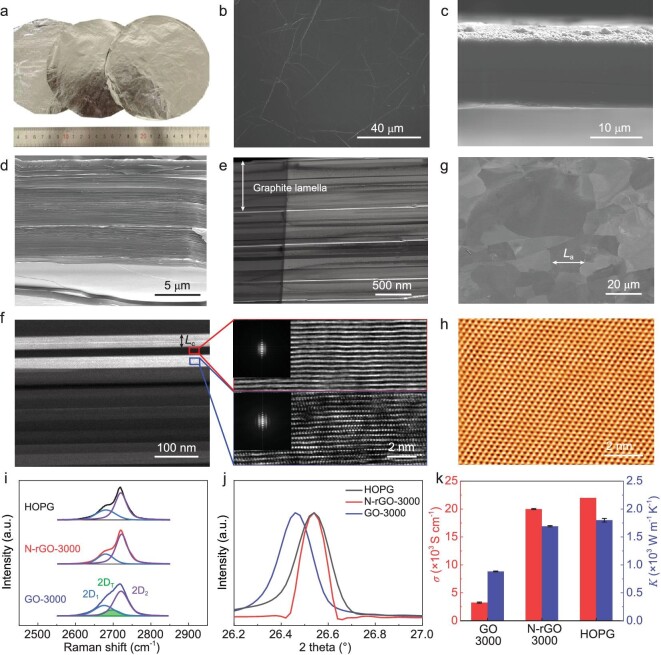
Structure and properties of large-area HOPG-like graphene films. (a) A photograph of three pieces of 10-μm-thick free-standing N-rGO-derived graphene films with a diameter of ∼14 cm. (b–d) SEM images of the surface (b) and cross section (c and d) of the films in (a). The cross-sectional samples in (c) and (d) were fabricated by ion beam cutting and tension fracture, respectively. (e) Cross-sectional BF-TEM image, where the parallel white lines are carbon-deficient interfaces of adjacent graphite lamellae (Supplementary Fig. 14). (f) Cross-sectional ADF-STEM image, in which the parallel bands of different brightnesses are single-crystal graphite grains with different orientations, as proven by HRTEM images and the corresponding Fourier transforms (insets). (g) SEM-ECC image of the film surface. (h) Atomically resolved in-plane STM image, showing a perfect graphite lattice. (i–k) Comparisons of Raman 2D peak (h), (002) XRD peak (i) and electrical and thermal conductivities (j) of GO- and N-rGO-derived graphene films and HOPG.

Using the high-temperature defect-enabled highly efficient graphitization approach, we fabricated large-area HOPG-like graphene films with N-rGO as precursor (Fig. 4a; Supplementary Figs 14 and 15). Such films have not been achieved so far by using either GO, rGO, a mixture of GO and rGO, graphene sheets, or CVD-grown large-area graphene films [6,12–15,21–23]. Due to the reduced oxygen content by hydrothermal treatment, N-rGO laminate undergoes weak structural expansion during annealing (Supplementary Fig. 9). As a result, the N-rGO-derived graphene films show a smooth surface and highly compact layered structure (Fig. 4b–d), forming a laminate of highly oriented submicrometer-sized graphite lamellae (average ∼700 nm) (Fig. 4e and Supplementary Fig. 14). The near absence of voids enables the film to have a high density of 2.22 g cm^−3^. These structural features are significantly different from those of the GO-derived graphene films, which feature abundant wrinkles, kinks and voids (Supplementary Figs 16 and 17). Although graphite lamellae are also formed in GO-derived graphene films, they are separated with voids and have much smaller thicknesses (average ∼200 nm) due to severe expansion during thermal annealing (Supplementary Fig. 8). Moreover, each graphite lamella in N-rGO-derived graphene films is composed of highly ordered graphite grain bands (Fig. 4f; Supplementary Figs 14 and 18). Meanwhile, well-distinguished large-size in-plane grains are also observed (Fig. 4g), and their perfect graphite lattice structure is confirmed by nanometer- and atomically resolved STM imaging (Fig. 4h; Supplementary Fig. 14). Notably, *L*_c_ and *L*_a_ are ∼50 nm and ∼20 μm (Fig. 4f and g, and Supplementary Figs 14 and 18), respectively, which are comparable to those of HOPG and approximately five and seven times larger than those of the GO-derived graphene films (Supplementary Figs 15 and 16). It is apparent that *L*_a_ is over 10 times larger than the lateral size of N-rGO precursors (∼1.5 μm), giving strong evidence of grain growth through interlayer migration and arrangement of carbon atoms, as discussed above.

Raman and XRD spectroscopy were further used to analyze the ordering of large-area N-rGO-derived graphene films. The invisible D peak suggests the restoration of graphitic sp^2^ structure (Supplementary Fig. 19), which is in good agreement with their high thermal stability (Supplementary Fig. 20). Importantly, they show a HOPG-like asymmetrical Raman 2D peak composed of only 2D_1_ and 2D_2_ subpeaks (Fig. 4i). Large-area Raman mappings of the intensity ratio of 2D_2_/(2D_2_ + 2D_T_) indicate predominant AB stacking with only ∼8.8% turbostratic-stacked regions (Supplementary Fig. 21). Accordingly, the film shows a (002) XRD peak with almost the same position as HOPG (Fig. 4j), which corresponds to a *d*-spacing of 3.3558 Å and a very low *P*_disorder_ of ∼6.8% [42] (Supplementary Table 2). In contrast, GO-derived graphene films have a high *P*_disorder_ of 28.7% and over 36.9% turbostratic-stacked regions (Fig. 4j, Supplementary Fig. 21 and Table 2).

For graphene-based materials, heat conduction mainly depends on the phonon transport from lattice vibrations of the sp^2^ network, and the electron transport is dominated by the delocalized π-bond over the graphene layer [10,11]. It has been reported that various lattice defects (e.g. vacancies, heteroatoms, GBs) and structural disorders (e.g. turbostratic stacking, voids, gaps, crumples), can form phonon- and electron-scattering centers [10,11], degrading the thermal and electrical properties. The large grain size, high crystallinity, remarkable ordering and AB stacking enable our HOPG-like graphene films to have superior electrical and thermal conductivities (Fig. 4k), ∼2.0 × 10^4^ S cm^−1^ and ∼1.7 × 10^3^ W m^−1^ K^−1^. These values are approximately six and two times higher than those of GO-derived films, respectively, and approach those of HOPG (∼2.2 × 10^4^ S cm^−1^ and ∼1.8 × 10^3^ W m^−1^ K^−1^) and single-crystal graphite (∼2.5 × 10^4^ S cm^−1^ and ∼2.0 × 10^3^ W m^−1^ K^−1^) [43].

Emerging 5G devices can provide ultrafast data transmission rates, more reliable and ubiquitous communications and ultralow latency, allowing various innovative services such as smart cities, the Internet of Things, artificial intelligence and satellite services [8,9]. However, the explosive growth of 5G devices, in particular mobile devices, has caused serious EMI issues that have detrimental impacts on not only device performance but also surrounding environments and human health [9]. Meanwhile, the significantly increased data transmission rate and highly integrated components in 5G mobile devices generate more heat, increasing the risk of overheating and failure of the devices [9]. Thus, a material that has superior EMI-SE and thermal conductivity at a minimal thickness is highly desired.

**Figure 5.**
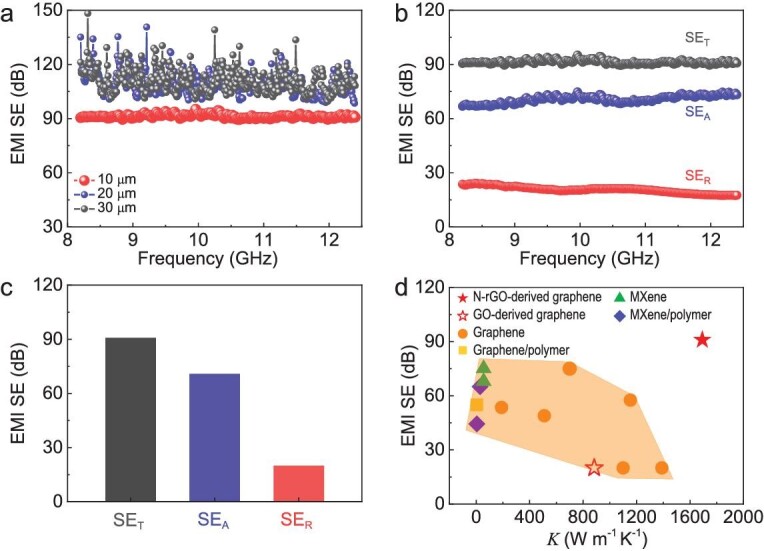
EMI shielding performances of HOPG-like graphene films. (a) EMI SE of graphene films with different thicknesses at X-band frequency range. (b and c) Total EMI SE (SE_T_), reflection SE (SE_R_) and absorption SE (SE_A_) of 10-μm-thick graphene films. (d) Comparison of EMI SE and thermal conductivities of 10-μm-thick graphene films with 10-μm-thick GO-derived graphene films and reported shielding materials (thickness ≥10 μm), including graphene- and MXene-based films (for details see Supplementary Table 4).

Ti_3_CNT_x_ MXene film is the best known EMI shielding material with EMI SE values of ∼75, 83, 97 and 116 dB at a thickness of 10, 20, 30 and 40 μm [44], respectively, but MXenes have a low thermal conductivity (smaller than 60 W m^−1^ K^−1^ [45]). We measured the EMI shielding performances of highly conductive N-rGO-derived graphene films with different thicknesses at X band from 8.2 to 12.4 GHz. Surprisingly, a superhigh EMI SE value of ∼90 dB is achieved for a 10-μm-thick film (Fig. 5a), which can block ∼99.9999999% of incident radiation, and the EMI SE exceeds the detection limit (∼100 dB) of the equipment when the thickness increases to 20 μm. Such performances are much better than those of GO-derived graphene films at the same thickness (Supplementary Fig. 22). Moreover, the shielding in our graphene film is dominated by absorption, which contributes ∼78% of the total EMI SE (Fig. 5b and c). The remarkable shielding performances of our graphene films are due to their superhigh electrical conductivity and the multiple internal reflections between graphene layers [2,44]. Significantly, N-rGO-derived graphene films not only have the highest EMI SE at a comparable thickness but also the highest thermal conductivity among the synthetic materials, including MXene- and graphene-based films (Fig. 5d and Supplementary Table 4), demonstrating their great potential for EMI shielding and thermal management in 5G mobile devices.

## CONCLUSION

Our work provides a new strategy to substantially accelerate the graphitization of graphene films by using N-doping to retain high-temperature defects, leading to significant improvements in their crystallinity, ordering, electrical and thermal conductivities, and EMI shielding performances. These findings pave the way for the practical application of graphene films to meet the rapid advancement of electronics and telecommunications. In principle, other heteroatoms—which can substitute the carbon atoms in a graphene lattice, hinder the restoration of defects and then be removed at elevated temperatures—could also be used to realize high efficiency graphitization. More generally, graphitization is the only method, known to date, for the crystallization of other types of carbon materials [16–20], including graphene fibers, CNT fibers, carbon fibers, polymer-derived graphite and HOPG. We envision that the defect-enabled highly efficient graphitization strategy should be applicable to the mass production of these carbon materials, and will greatly improve their electrical and thermal properties for various applications. However, the atomic-level mechanism of N-doping and lattice defects for boosting graphitization of graphene films needs further investigation, especially by combining large-scale molecular dynamic simulations with *in-situ* experiments such as TEM observations, which is essential for precise control of the structure of graphene films.

## Supplementary Material

nwad147_Supplemental_File
