# Supplementary material for: Defects boost graphitization for highly conductive graphene films
Source: Natl Sci Rev. 2023 May 19;10(7):nwad147. doi: 10.1093/nsr/nwad147 (PMC10319761; doi:10.1093/nsr/nwad147)
Supplement: nwad147_Supplemental_File [file nwad147_supplemental_file.pdf]

Supplementary information

Defects boost graphitization for highly conductive graphene films

Qing Zhang[1,2], Qinwei Wei[1,2], Kun Huang[1,2], Zhibo Liu[1,2], Wei Ma[1,2], Zehui Zhang[3], Yanfeng Zhang[3], Hui-Ming Cheng[1,2,4] and Wencai Ren[1,2,*]

[1]Shenyang National Laboratory for Materials Science, Institute of Metal Research, Chinese Academy of Sciences, Shenyang 110016, China;

[2]School of Materials Science and Engineering, University of Science and Technology of China, Shenyang 110016, China;

[3]School of Materials Science and Engineering, Peking University, Beijing 100871, China;

[4]Shenzhen Institute of Advanced Technology, Chinese Academy of Sciences, Shenzhen 518055, China

*Corresponding author. E-mail: wcren@imr.ac.cn

MATERIALS AND METHODS

Synthesis of GO, N-rGO and rGO sheets and dispersions. GO sheets were synthesized following the modified Hummers' method, and N-rGO sheets were synthesized by hydrothermal treatment of GO sheets with the addition of ammonia [26]. During hydrothermal treatment process, 1.5 L GO aqueous dispersion (0.22 g L^{-1}) was put into 2 L Teflon stainless steel autoclaves, and 5 mL of ammonia was

added to adjust pH within a certain range of 8-9, then heated at 150 ℃ (120 ℃) for 16 h (8 h) to obtain the dispersions of 10.3 wt% N-rGO (9.3 wt% N-rGO) sheets. The dispersion of rGO sheets was synthesized by hydrothermal treatment of GO sheets in water without the addition of ammonia at 150 ℃ for 8 h.

Synthesis of graphene films. As-synthesized N-rGO or rGO dispersion (4.5 L) was assembled into a film by compression filtration at 6 bar on polyether sulfone (PES) microfiltration membrane. For comparison, GO dispersion containing 1 g GO sheets was also assembled into a film with the same method. The diameter of the films was ~14 cm which was determined by the diameter of the pressure-driven filtration tank. The as-assembled wet-state films were sandwiched by two pieces of sponges, and dried on a hot plate at 50 ℃ with mild pressing. After drying, the films were peeled off from PES membrane and kept in oven at 100 ℃ to remove unbound water and NH_3 for further thermal treatment and structural characterizations. To fabricate graphene films, the N-rGO, GO and rGO films were heated to 3000 ℃ in argon flow (8000 sccm) in a graphitization furnace (Zhuzhou Chenxin Induction Equipment Co., Ltd, CX-GF20/30VT) within 420 min, then kept graphitization for 10 minutes, and finally naturally cooled down to room temperature in Ar flow (6000 sccm). After that, a cold-pressing at 30 MPa was carried out for 5 minutes to remove the voids and obtain highly compact graphene films, i.e., N-rGO-, GO- and rGO-derived graphene films. In our experiments, all the graphene films obtained after 3000 ℃ annealing were measured at a similar thickness of ~10 μm by a micrometer caliper. It should be pointed out that the GO films were preheated at 200 ℃ for 5 h in air oven before the

above annealing process, in order to avoid the explosion caused by violent gas release from the rapid decomposition of oxygen functional groups during the following high-temperature annealing.

To reveal the structure and properties evolution during graphitization process, in addition to the above films obtained at 3000 ℃, the N-rGO and GO films were heated to three different temperatures (2000, 2500 or 2800 ℃) in argon flow and kept for 0.5 h for each temperature, and then natually cooled down to room temperature followed by cold-pressing at 30 MPa for 5 min. To investigate the expansion occured during the whole annealing process, the N-rGO and GO films annealed at seven different temperatures (100, 1000, 1400, 2000, 2500, 2800 and 3000 ℃) were synthesized and no further cold-pressing was used before characterizations for such samples. Thermal annealing at no higher than 1400 ℃ was carried out in a tube furnace (Lindberg Blue MTF55030C), and a graphitization furnace (Zhuzhou Chenxin Induction Equipment Co., Ltd, CX-GF20/30-VT) was used for annealing at higher than 1400 ℃.

Chemical composition analyses. The elemental composition was characterized by elemental analyzer (vario MACRO cube of Elementar), which worked with high-temperature combustion and gas separation method to achieve precise quantification of elemental composition. The chemical states of C, O and N species were characterized by XPS on ESCALAB 250 using monochromatic Al Kα radiation, and all spectra were calibrated to the binding energy of C=C bonds (284.6 eV).

XRD measurements and analyses. XRD patterns of the graphene films were collected by a XRD system (Rigaku, SmartLab D/teX Ultra 250 using Cu Kα radiation).

The *d*-spacing (*d*) of graphene films was estimated by the Bragg's equation based on the XRD (002) peak:

$$n\lambda = 2d\,sin\theta \qquad (1)$$

where λ is the X-ray wavelength (0.15406 nm), and θ (rad) is the scattering angle.

To quantify the degree of ordering of graphene films, P_{disorder} (p), was estimated using Bacon's equation [42]:

$$d_{002} = 3.440 - 0.086\,(1 - p) - 0.064p\,(1 - p) \qquad (2)$$

To make a comparison with the L_c derived from ADF-STEM observations, Scherrer equation was also used to estimte L_c [34,35]:

$$L_c = \frac{K\lambda}{\beta \cos\theta} \qquad (3)$$

where K is a constant (here, 0.89), λ (nm) is the X-ray wavelength, θ (rad) is the scattering angle, and β is the full width at half maximum (FWHM) of the (002) peak without eliminating the influence of instrumental broadening.

Raman measurements for defects and stacking order analyses. Raman spectra were acquired on LabRAM HR800 (laser wavelength λ_L = 532 nm). The laser spot was ~1 μm and the laser power on the sample surface was kept below 15 mW to avoid heating effect and structural damages. Raman mapping was performed with a step of 1 μm.

We performed Lorentzian fitting on the 2D peaks to evaluate the fraction of AB-stacking based on the intensity ratio of $2D_2/(2D_2 + 2D_T)$ [28,29]. The proportion of turbostratic-stacked regions in large-area GO- and N-rGO-derived graphene films was analyzed from the Raman mappings of intensity ratio of $2D_2/(2D_2 + 2D_T)$, which contain 5751 spectra for each sample. The intensity ratio of $2D_2/(2D_2 + 2D_T)$ below 0.99 was considered as turbostratic-stacked regions.

Thermal stability analyses. Thermogravimetric-differential thermal analysis (TG-DTA) measurements were carried out on Netzsch STA-499C. The samples with similar thickness and shape were heated up to 1100 ℃ at a heating rate of 10 ℃ min^{-1} in air atmosphere. The temperature corresponding to the maximum mass decomposition rate (T_{max}) reflects the difference of the crystalline quality of the graphene films.

SEM characterizations. The surface and cross-sectional morphology of graphene films were characterized by SEM (Verios G4 UC, 10 kV). The cross-sectional SEM samples were fabricated by ion beam cutting (Leica, EM TIC 3X) or tension fracture. The former samples were used for characterizing the voids that could not be removed by pressing, i.e., the degree of compaction. The latter samples were mainly used for observing the microstructure of films after annealing at different temperatures (e.g., film expansion). The in-plane grain structure and L_a of the graphene films were characterized by SEM-ECC imaging, where the grains with different orientations show different brightnesses because of the different backscattered electron densities [19]. To do that, the samples were peeled off by scotch tape to obtain fresh and

smooth surface. Over twenty-five grains were measured to give the average values of L_a for each sample.

TEM characterizations. Focused ion beam cutting (FEI LD Helios G5 UX) was used to fabricate cross-sectional TEM samples. Before cutting, the films obtained after annealing were cold-pressed at 30 MPa for 5 min. The cross-sectional microstructure of graphene films was characterized by TEM (FEI Talos F200X). The through-plane grain structure was identified based on the diffraction contrast in ADF-STEM images. The camera length and beam convergence angle were 98 mm and 10.5 mrad, respectively. The collective angle ranges for ADF- and BF-STEM were 12-24 mrad and 9 mrad, respectively. Duing ADF-STEM measurements, the cross-sectional samples were rotated finely around the through-plane direction of graphene films to distinguish the adjacent grains with a small rotation angle, as shown in Supplementary Fig. 18. Over twenty-five grains were measured to give the average values of L_c for each sample. HRTEM images and selected area electron diffraction (SAED) patterns were also measured to characterize the degree of ordering and crystallinity of the graphene films.

STM characterizations. The atomically-resolved in-plane structure of GO and N-rGO films after annealing at different temperatures was characterized by STM (Omicron ultra-high-vacuum RT-STM), under a base pressure better than 10^{-10} mbar at room temperature. STM imaging was accomplished in the constant current mode (variable height) using chemically etched W tips with typical tunneling parameters of

100-2000 mV (bias voltage) and 3.33 nA (tunneling current). Before testing, the samples were peeled off by scotch tape to expose fresh surfaces.

Electrical and thermal conductivity characterizations. To measure the electrical and thermal conductivities, the films were cut into disks with a radius (r) of 12.7 mm. The thickness (h) was kept at ~10 μm, measured by both micrometer caliper and cross-sectional SEM. The electrical conductivity (σ) was measured using a four-point probe system (RTS-9) at room temperature. The in-plane thermal conductivity (K) was calculated from the equation $K = \alpha \times c \times \rho$, where α, c, and ρ represent the in-plane thermal diffusivity, specific heat capacity, and density of the film, respectively. The in-plane thermal diffusivity was measured by LFA467 Hyper Flash (IR detector: InSb; sample holder: In-plane round/25.4 mm diameter; test temperature: 25 ℃; laser voltage: 180 V; pulse width: 50 μs; calculation model: In-plane isotropy + pulse correction). The density was obtained from the equation $\rho = m/\pi r^2 h$, where m is the mass of the film measured with an electronic balance (Mettler Toledo, XSR225DU/AC, 0.01 mg). For each type of graphene film, five samples were measured and each sample were measured for five times to obtain the average electrical and thermal conductivities.

EMI shielding performance measurements. The EMI SE was measured by PAN Network Analyzer (N5244A) in X-band frequency range of 8.2-12.4 GHz. The samples were cut into a rectangular shape (22.9 mm × 10.2 mm) and sandwiched between the waveguide sample holders. EMI shielding efficiency represents the

ability to block incident waves in terms of percentage. EMI SE [dB] was converted into EMI shielding efficiency [%] using the equation:

$$\text{Shielding efficiency } (\%) = 100 - \left(\frac{1}{10^{\frac{SE}{10}}}\right) \times 100 \quad (5)$$

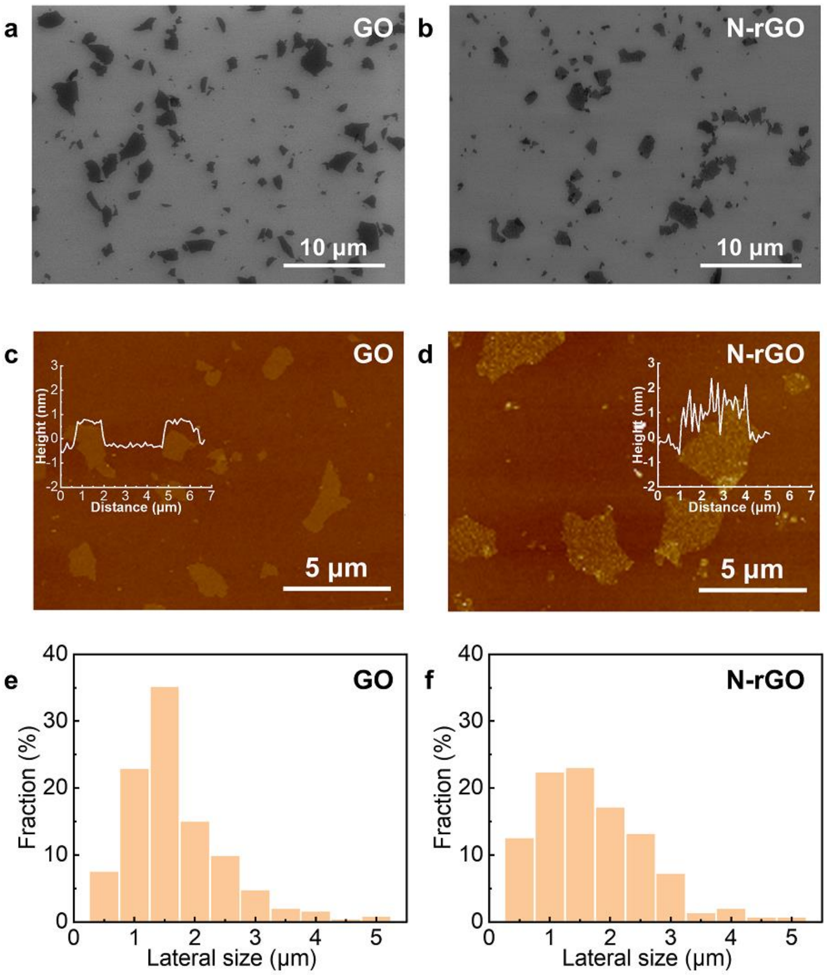

Supplementary Figure 1. Characterizations of GO and N-rGO sheets. a, b, SEM images of GO (**a**) and N-rGO (**b**) sheets deposited on SiO_2/Si wafer. **c**, **d**, AFM images of GO (**c**) and N-rGO (**d**) sheets. Insets: Height profiles showing that the sheets are monolayers with a thickness of ~1.0 nm. **e, f**, Lateral size distributions of GO (**e**) and N-rGO (**f**) sheets. Note that the thickness and lateral size are almost unchanged after hydrothermal reduction with ammonia.

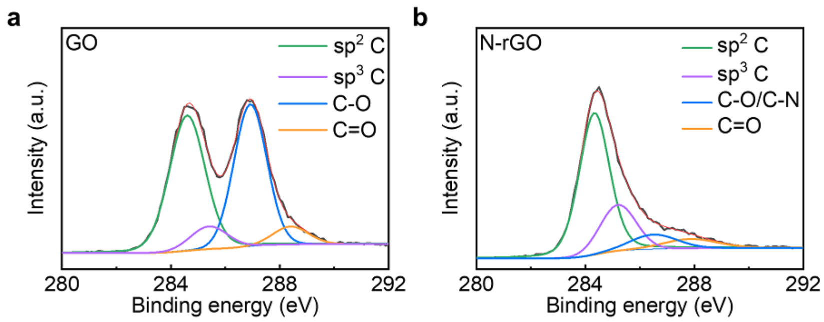

Supplementary Figure 2. C1s XPS spectra of GO and N-rGO. a, GO. **b,** N-rGO. They clearly show the significant decrease of O content in N-rGO compared to GO.

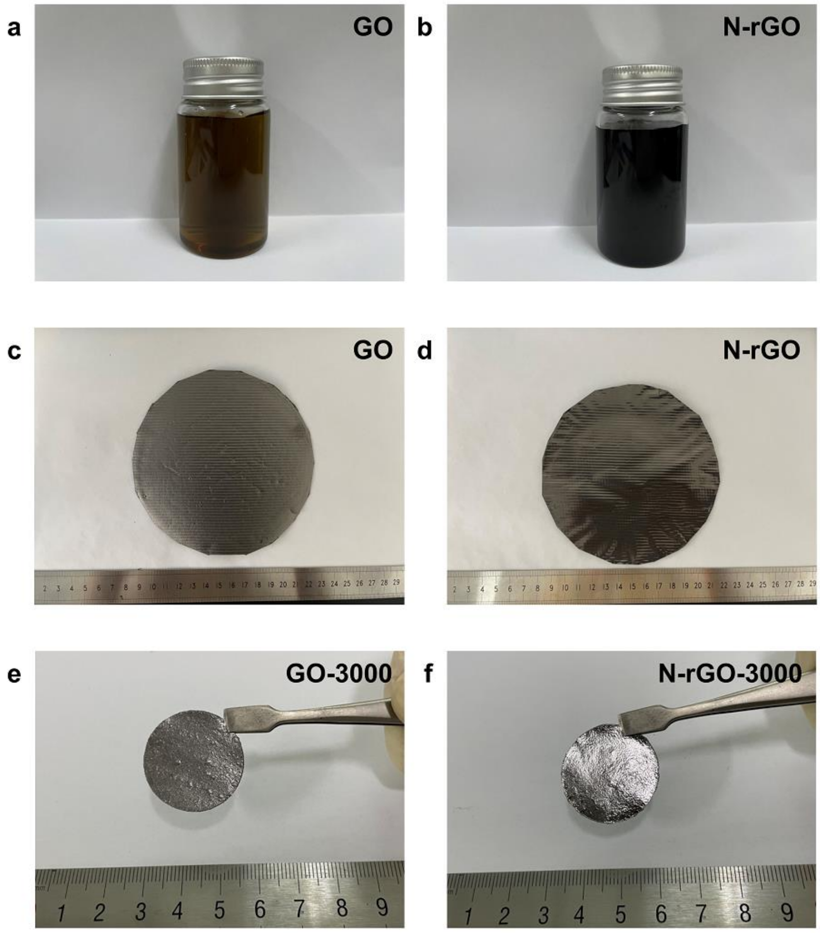

Supplementary Figure 3. Photographs of GO and N-rGO dispersions, laminates and derived graphene films. a, **b**, As-synthesized GO (**a**) and N-rGO (**b**) aqueous dispersions. **c**, **d**, GO (**c**) and N-rGO (**d**) laminates obtained by filtration. **e, f**, GO-derived (**e**) and N-rGO-derived (**f**) graphene films obtained under 10-min graphitization at 3000 ℃ followed by cold pressing. A small mold with a radius of 12.7 mm was used for pressing, in order to meet the requirements for thermal conduction tests.

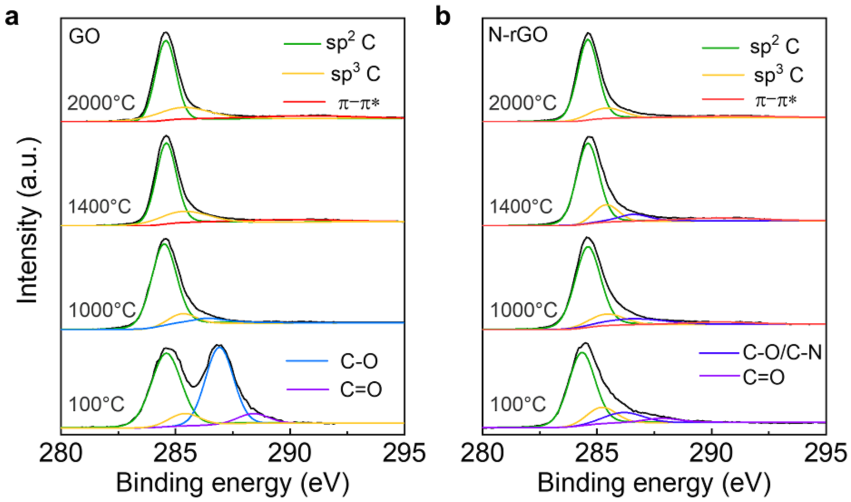

Supplementary Figure 4. C1s XPS spectra of as-synthesized GO (a) and N-rGO (b) films and after annealing at 1000 ℃, 1400 ℃, and 2000 ℃.

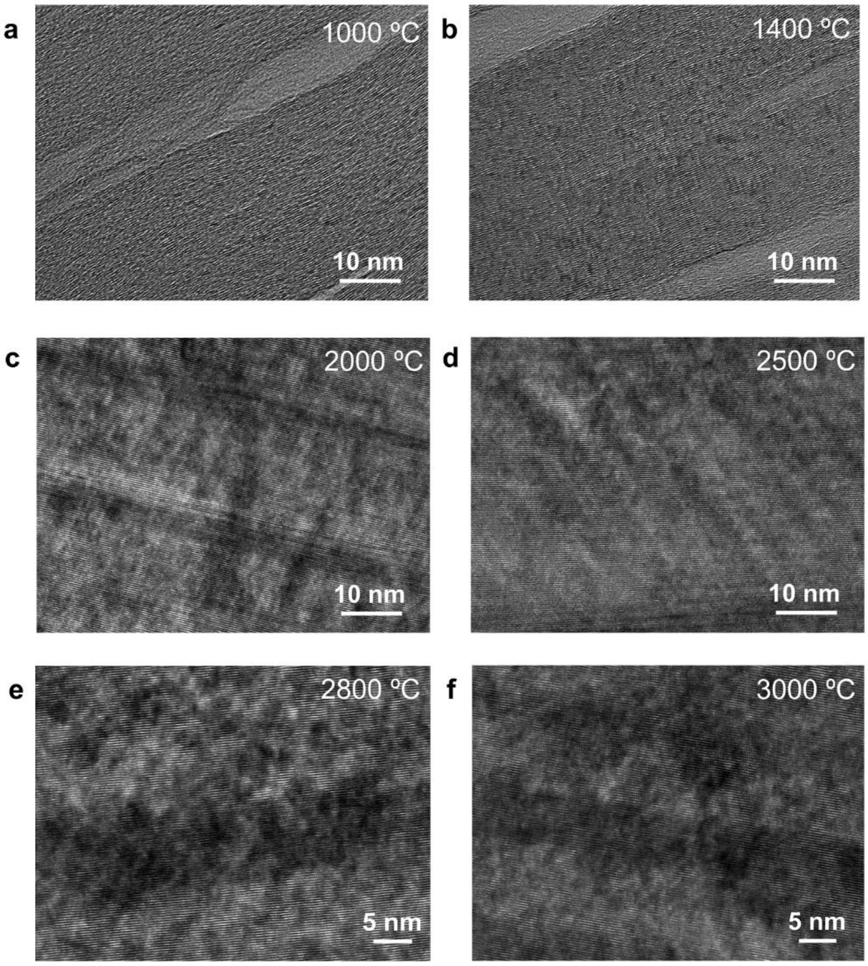

Supplementary Figure 5. Structural evolution of GO films upon thermal annealing. a-f, HRTEM images of GO films after annealing at 1000 ℃ (**a**), 1400 ℃ (**b**), 2000 ℃ (**c**), 2500 ℃ (**d**), 2800 ℃ (**e**) and 3000 ℃ (**f**).

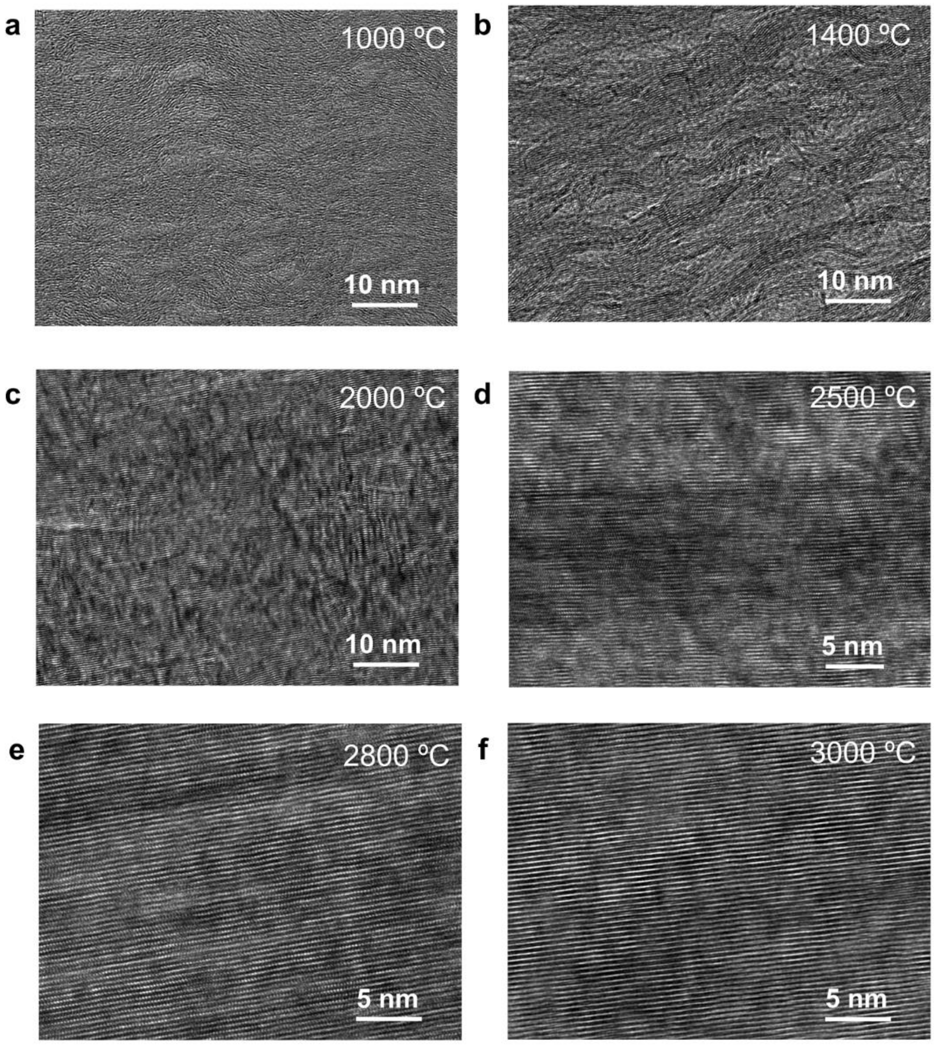

Supplementary Figure 6. Structural evolution of N-rGO films upon thermal annealing. a-f, HRTEM images of N-rGO films after annealing at 1000 ℃ (**a**), 1400 ℃ (**b**), 2000 ℃ (**c**), 2500 ℃ (**d**), 2800 ℃ (**e**), 3000 ℃ (**f**).

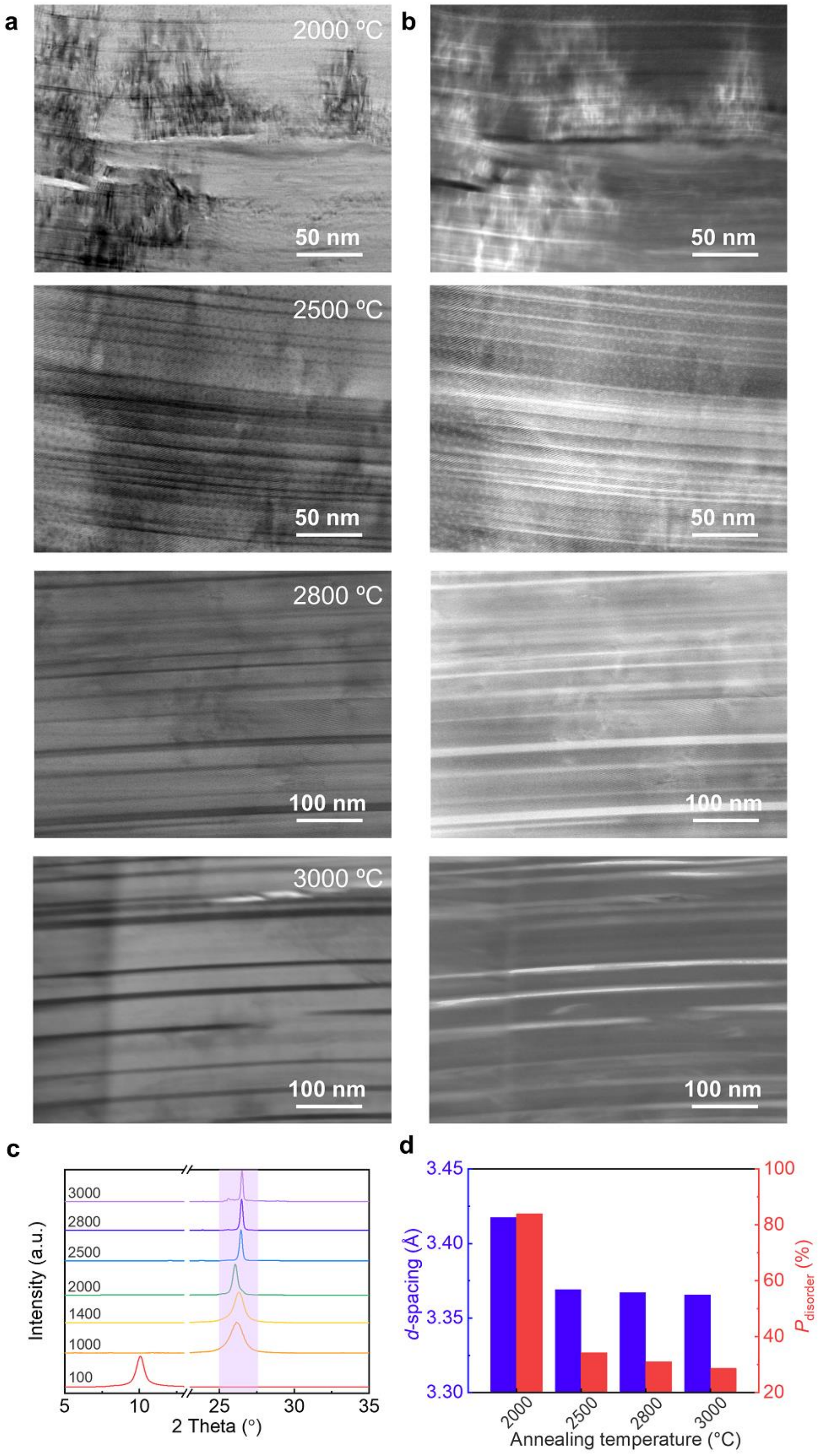

Supplementary Figure 7. Structure evolution of GO film during thermal annealing. a,b, Cross-sectional BF-STEM images (**a**) and ADF-STEM images (**b**) of GO films obtained after annealing at 2000 ℃, 2500 ℃, 2800 ℃ and 3000 ℃, respectively. **c**, **d**, XRD (002) peak evolution of GO films with annealing temperature

(**c**) and the calculated *d*-spacing and $P_{disorder}$ based on the XRD (002) peak position (**d**).

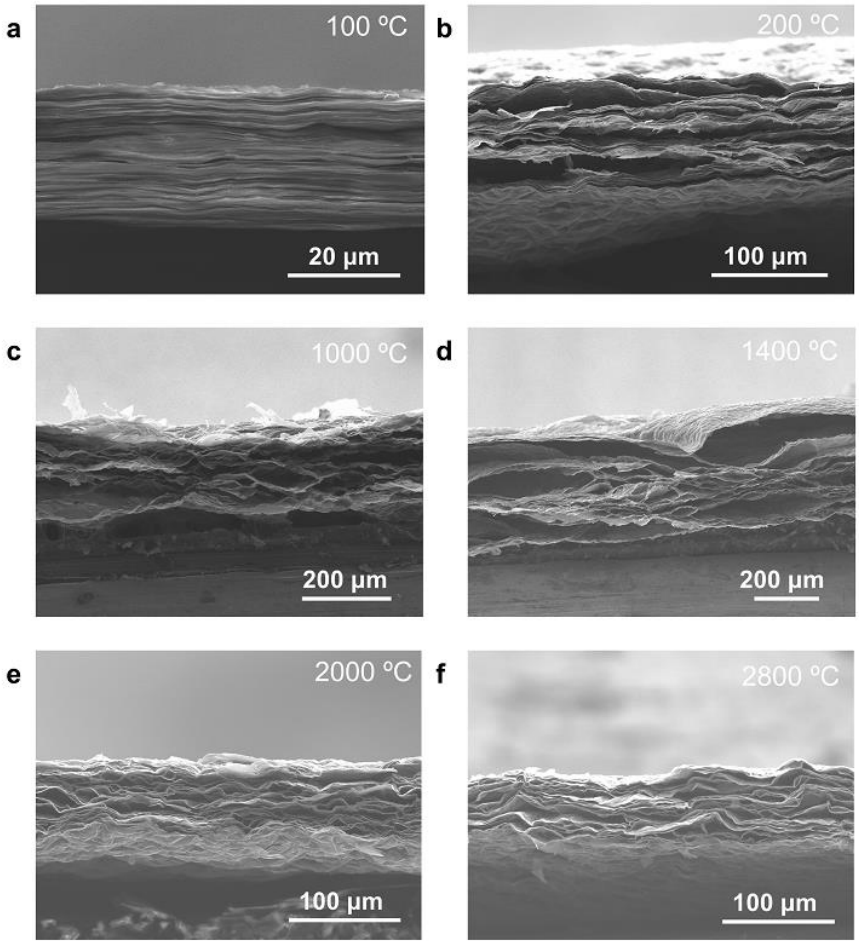

Supplementary Figure 8. Morphology evolution of GO film with annealing temperature. Cross-sectional SEM images of the as-synthesized GO film (**a**) and after annealing at 200 ℃ (**b**), 1000 ℃ (**c**), 1400 ℃ (**d**), 2000 ℃ (**e**), and 2800 ℃ (**f**) for 0.5h for each temperature. In order to see the laminate structure change during annealing, no pressing was conducted after annealing for all the films to avoid the destruction on the expansion and delamination structure.

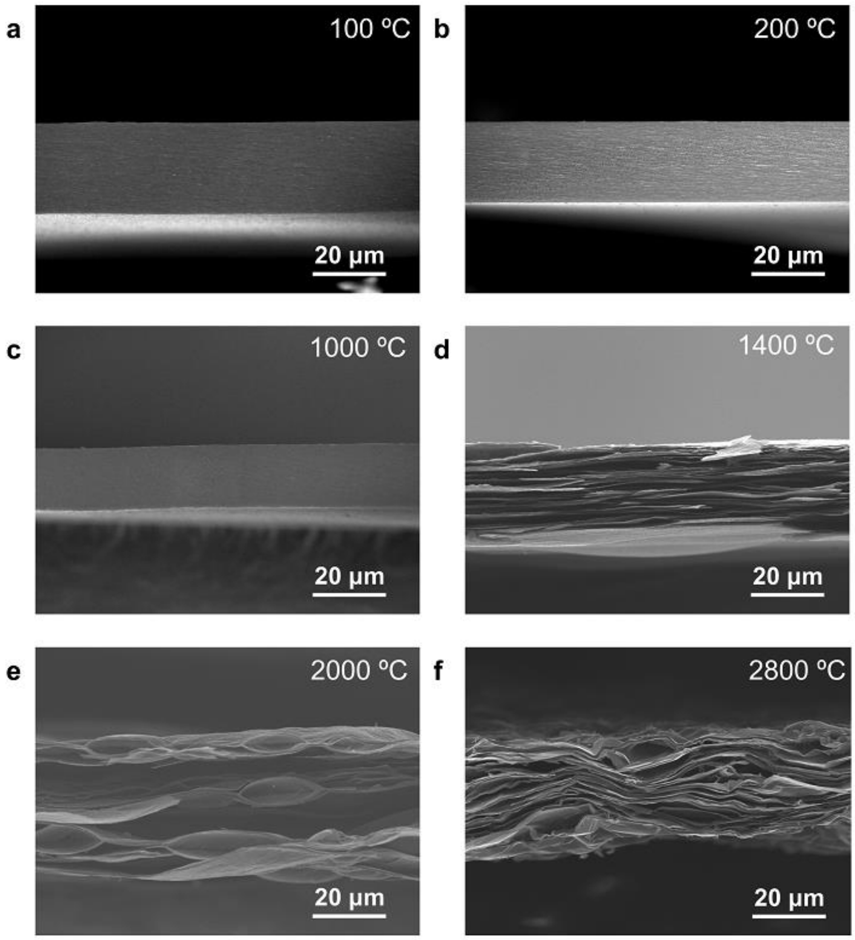

Supplementary Figure 9. Morphology evolution of N-rGO film with annealing temperature. Cross-sectional SEM images of the as-synthesized N-rGO film (**a**) and after annealing at 200 ℃ (**b**), 1000 ℃ (**c**), 1400 ℃ (**d**), 2000 ℃ (**e**), and 2800 ℃ (**f**) for 0.5 h for each temperature. In order to see the laminate structure change during annealing, no pressing was conducted after annealing for all the films to avoid the destruction on the expansion and delamination structure.

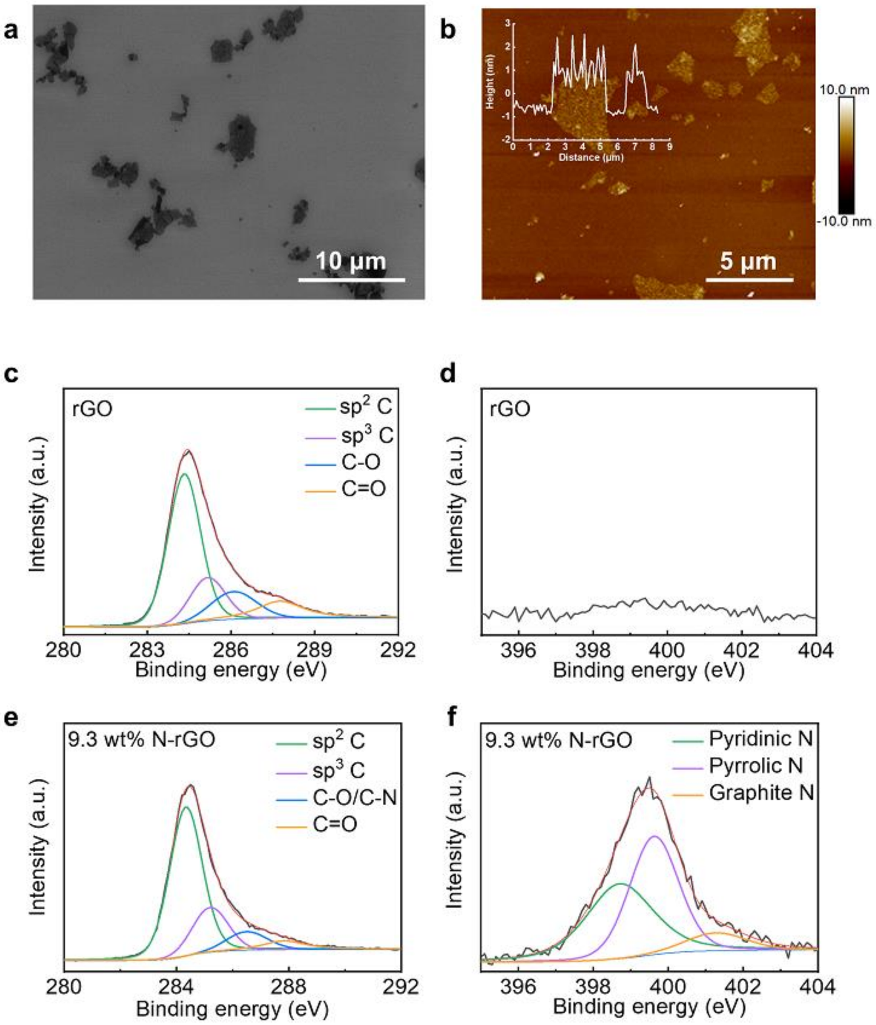

Supplementary Figure 10. Characterizations of rGO sheets and 9.3 wt% N-rGO. **a**, SEM image of rGO sheets. **b**, AFM image and the height profile of rGO sheets (inset). **c**, **d**, C1s (**c**) and N1s (**d**) XPS spectra of rGO. **e**, **f**, C1s (**e**) and N1s (**f**) XPS spectra of 9.3 wt% N-rGO.

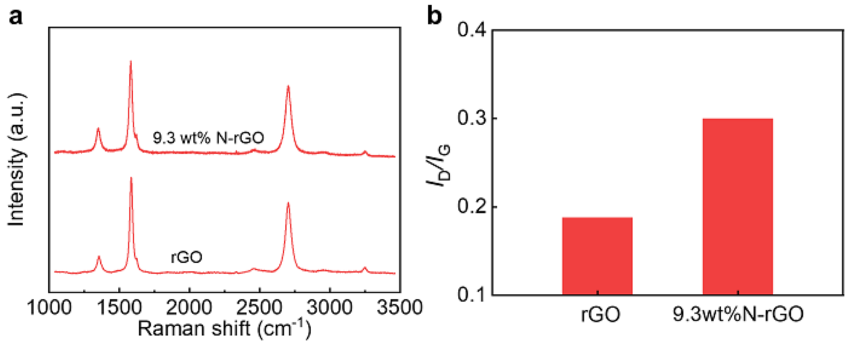

Supplementary Figure 11. Comparison of the Raman spectra (a) and corresponding I_D/I_G (b) of rGO and 9.3 wt% N-rGO films after thermal annealing at 2000 ℃.

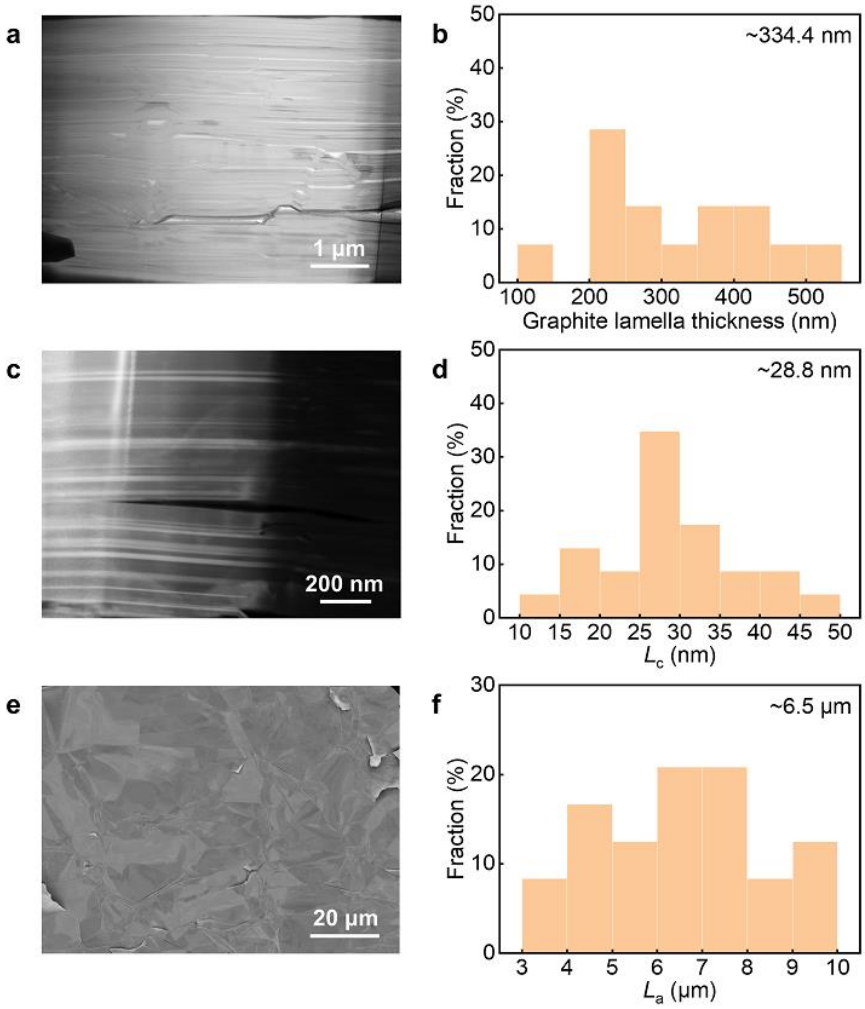

Supplementary Figure 12. Structure characterizations of 9.3 wt% N-rGO-derived films. **a**, **b**, Cross-sectional low-magnification BF-TEM image (**a**) and graphite lamella thickness distribution (**b**). **c**, **d**, Cross-sectional ADF-STEM image (**c**) and L_c distribution (**d**). **e**, **f**, SEM-ECC image (**e**) and L_a distribution (**f**).

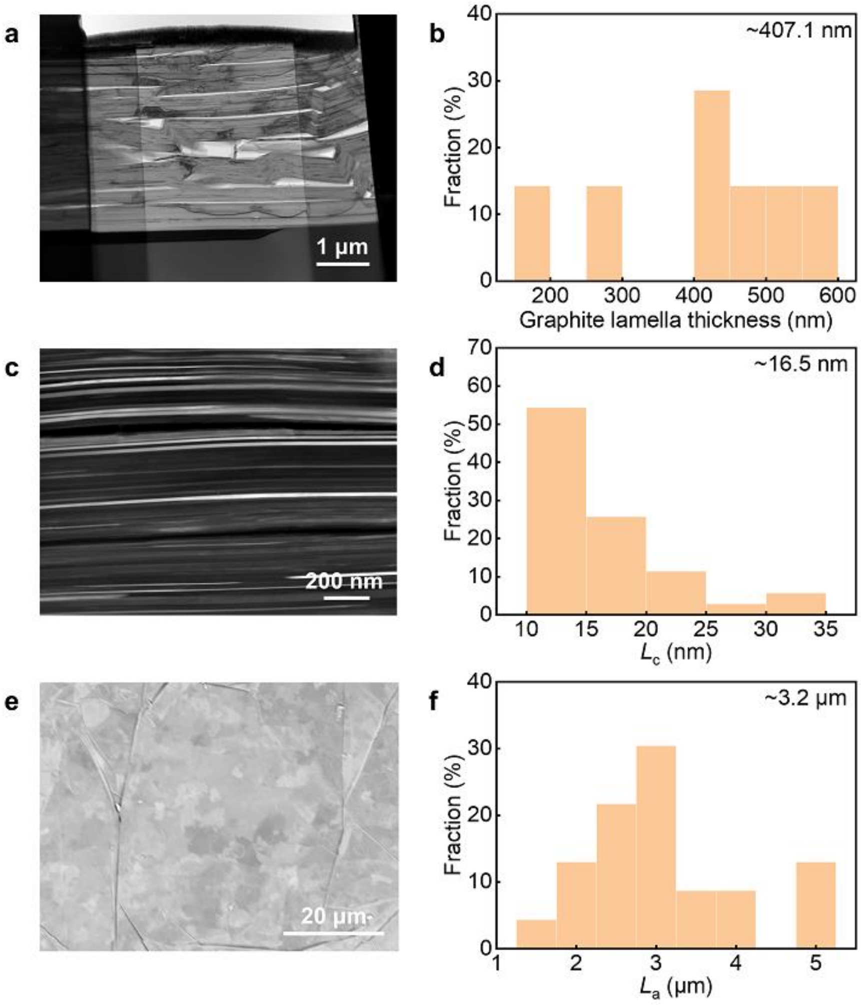

Supplementary Figure 13. Structure characterizations of rGO-derived graphene films. **a**, **b**, Cross-sectional low-magnification BF-TEM image (**a**) and graphite lamella thickness distribution (**b**). **c**, **d**, Cross-sectional ADF-STEM image (**c**) and L_c distribution (d). **e**, **f**, SEM-ECC image (**e**) and L_a distribution (**f**).

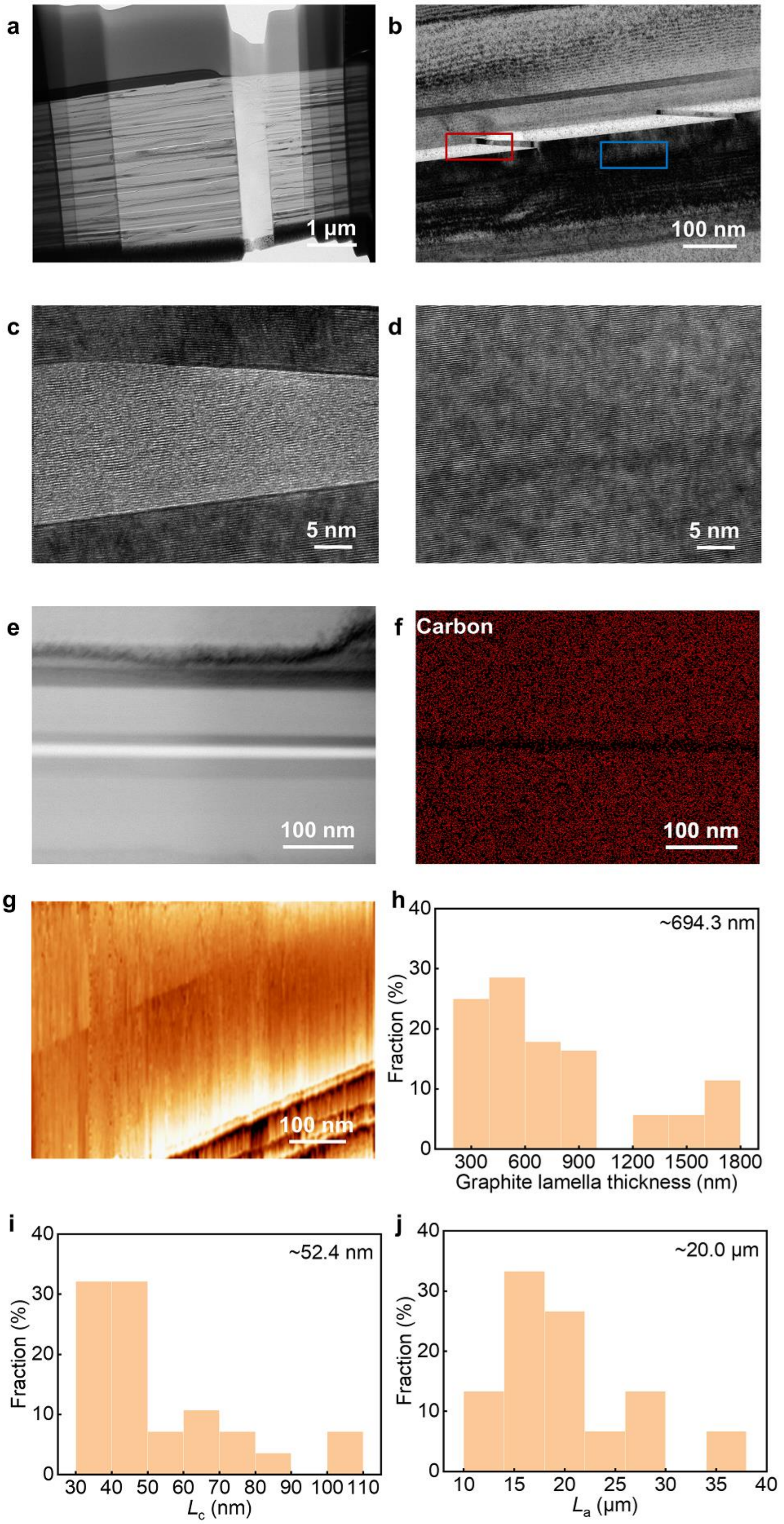

Supplementary Figure 14. Structure characterizations of N-rGO-derived graphene films. a,**b**, Cross-sectional low-magnification (**a**) and high-magnification

(**b**) BF-TEM images, showing highly oriented laminate crystalline structure. **c**,**d**, HRTEM images of the regions indicated by red box (**c**) and blue box (**d**) in b, respectively, showing the highly ordered crystalline structure and interface (white line in b). **e**,**f**, BF-STEM image (**e**) and the corresponding STEM-EDX elemental mapping (**f**), indicating that the interface is carbon-deficient. **g**, Nanometer-STM image. **h-j.** Graphite lamella thickness (**h**), L_c (**i**) and L_a (**j**) distributions obtained from BF-TEM, ADF-STEM and SEM-ECC measurements, respectively.

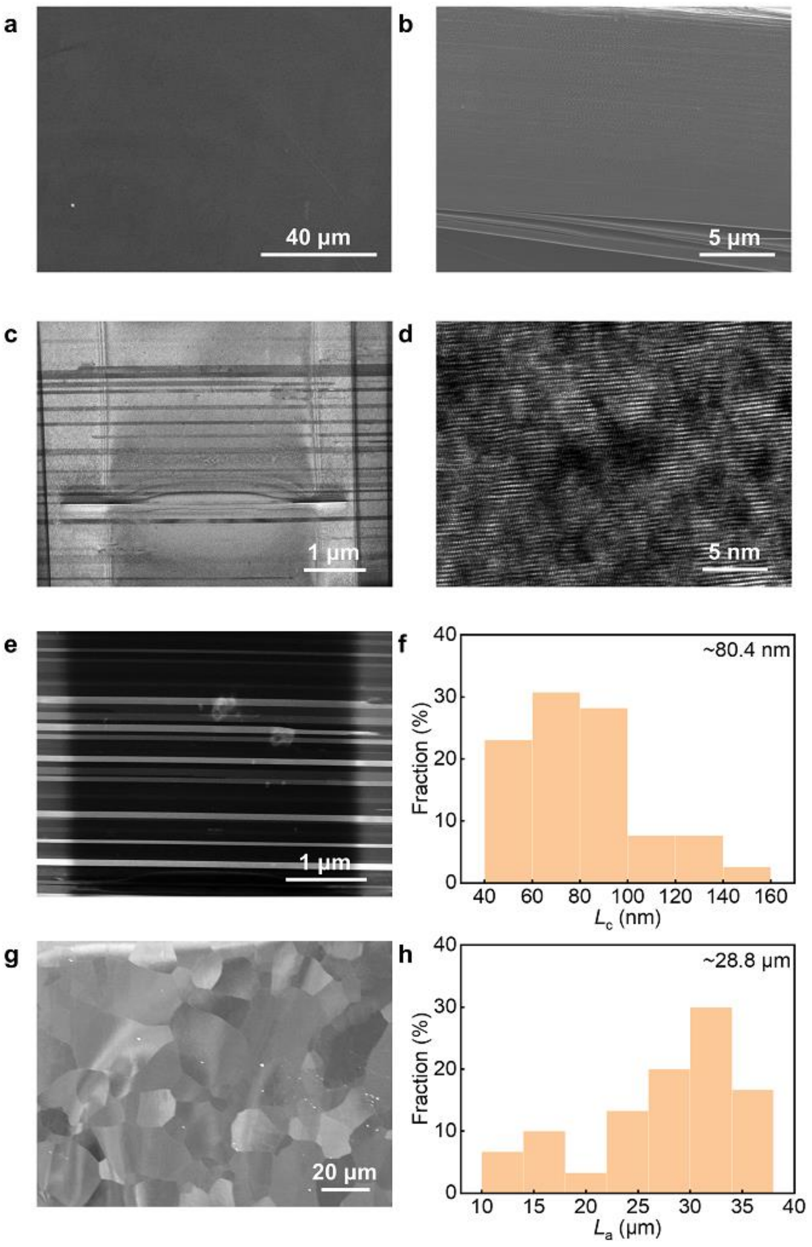

Supplementary Figure 15. Structure characterizations of HOPG. **a**,**b**, SEM images of the surface (**a**) and cross section (**b**) of HOPG. The cross-sectional samples were fabricated by fracture tension. **c**,**d**, Cross-sectional BF-TEM (**c**) and HRTEM (**d**) images, showing highly oriented laminate structure of high crystallinity. **e**,**f**, ADF-STEM image (**e**) and the corresponding L_c distributions (**f**). **g**,**h**, SEM-ECC image of the surface (**g**) and the corresponding L_a distribution (**h**). For surface observations, the samples were peeled off by scotch tape to expose fresh surfaces.

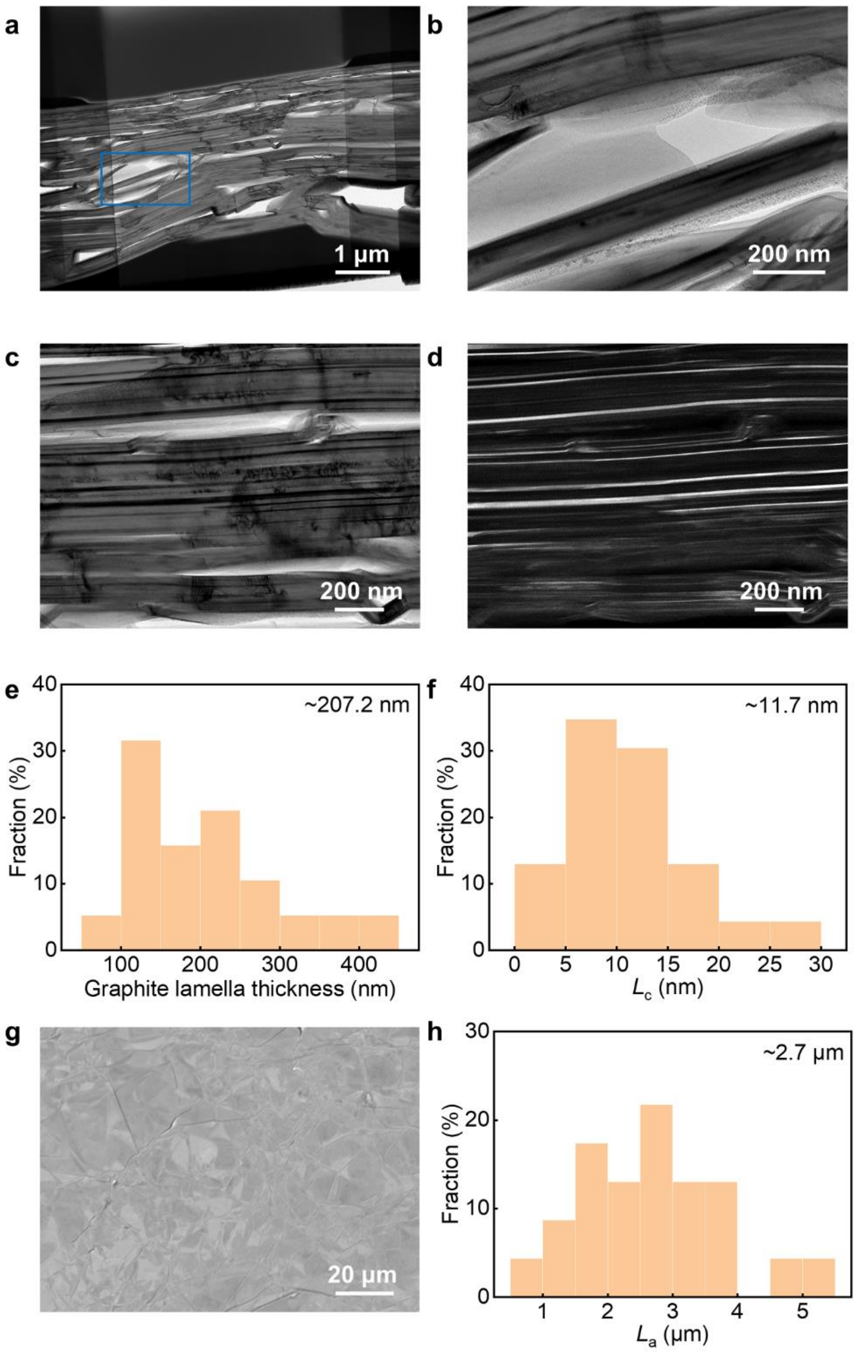

Supplementary Figure 16. Structure characterizations of GO-derived graphene films. **a**, Cross-sectional BF-TEM image, showing many voids, kinks and folds. **b**, HRTEM image of the region indicated by blue box in **a**, showing amorphous carbon filled in the voids. **c**, **d**, Cross-sectional BF-TEM image (**c**) and the corresponding ADF-STEM (**d**) image of the grain structure, showing that it is composed of very narrow grain bands with L_c of ~12 nm. **e**, **f**, Graphite lamella thickness (**e**) and L_c (**f**) distributions obtained from BF-TEM and ADF-STEM measurements, respectively. **g**, **h**, SEM-ECC image (**g**) and the corresponding L_a distribution (**h**).

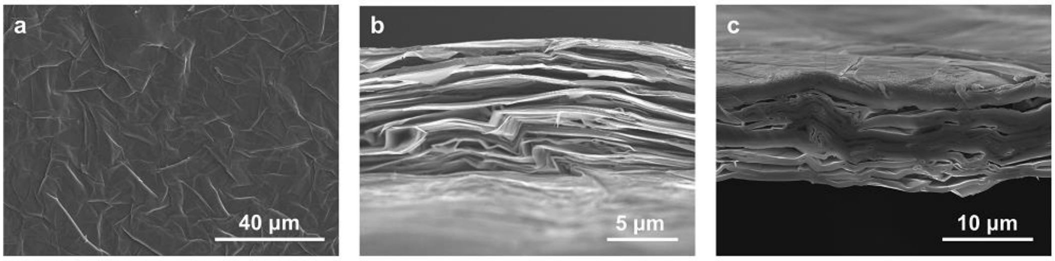

Supplementary Figure 17. Morphology of GO-derived graphene films. a-c, SEM images of the surface (**a**) and cross section (**b**,**c**) of GO-derived graphene films, showing abundant wrinkles and voids. The samples were peeled off by scotch tape to expose fresh surfaces for surface observations, and the cross-sectional samples in b and c were fabricated by tension fracture and ion beam cutting, respectively.

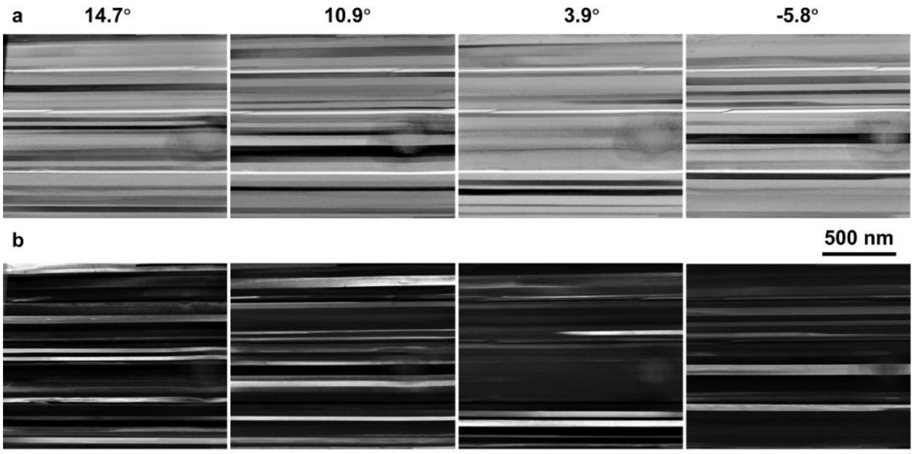

Supplementary Figure 18. Cross-sectional STEM image of the N-rGO-derived graphene film. BF-STEM images (**a**) and the corresponding ADF-STEM images (**b**) of the film at different angle of rotation.

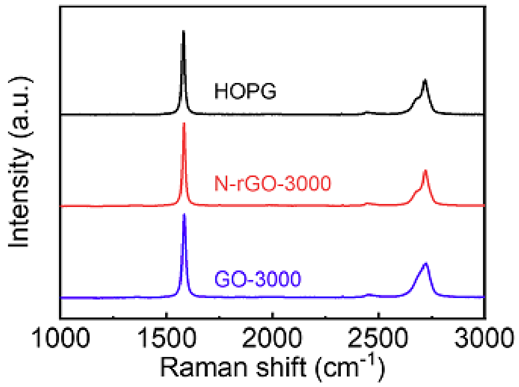

Supplementary Figure 19. Comparisons of Raman spectra of GO- and N-rGO-derived graphene films and HOPG.

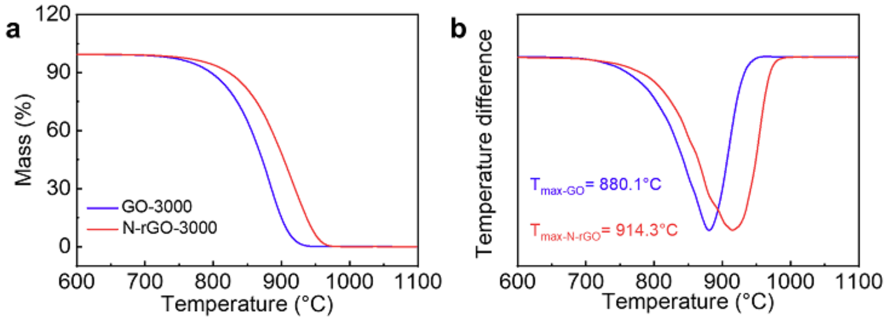

Supplementary Figure 20. Thermal stability of GO-3000 and N-rGO-3000 films in air atmosphere. TGA (**a**) and the corresponding normalized DTA (**b**) curves. The increased thermal stability of graphene films indicates their improved crystallinity.

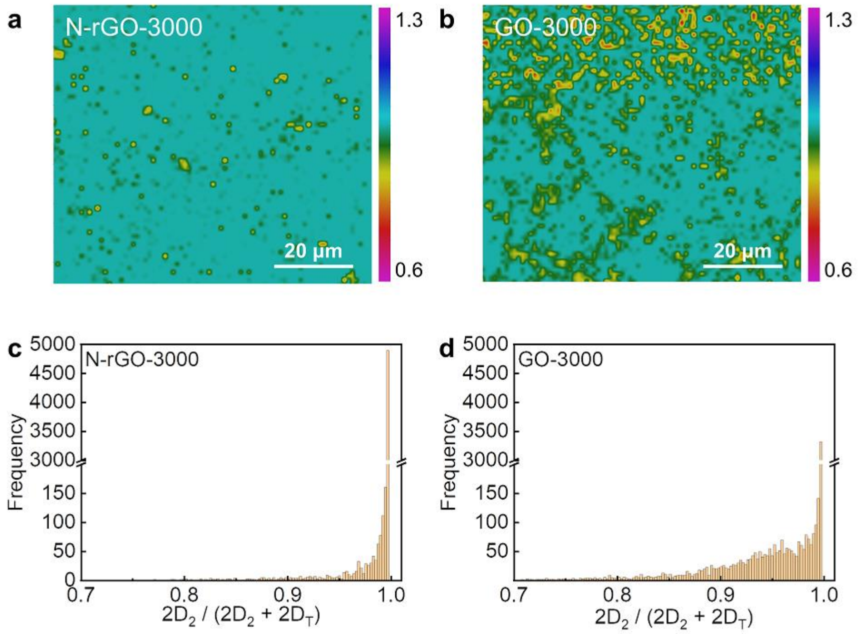

Supplementary Figure 21. Evaluation on the proportion of turbostratic-stacked regions in N-rGO- and GO-derived graphene films. a,b, Raman mappings of the intensity ratio of $2D_2/(2D_2 + 2D_T)$ of N-rGO- (**a**) and GO-derived (**b**) graphene films. **c,d,** $2D_2/(2D_2 + 2D_T)$ intensity ratio distributions of N-rGO- (**c**) and GO-derived (**d**) graphene films, which were extracted from the Raman mappings. The intensity ratio of $2D_2/(2D_2 + 2D_T)$ below 0.99 was considered as turbostratic-stacked regions.

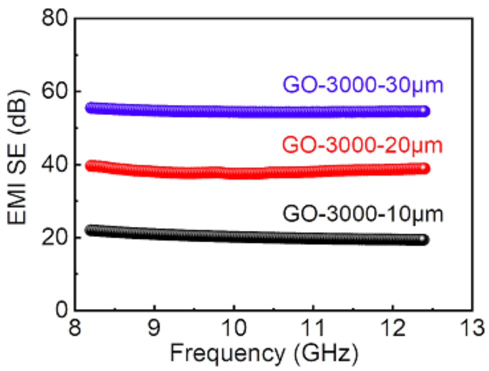

Supplementary Figure 22. EMI SE of GO-derived graphene films with different thicknesses at X-band frequency range.

Supplementary Table 1. Elemental analyses of GO, N-rGO, rGO sheets and N-rGO-derived graphene films.

Sample	Carbon (wt%)	Oxygen (wt%)	Nitrogen (wt%)	C/O ratio	C/N ratio
GO	48.82	46.60	1.53	1.40	37.22
GO-1000	92.04	7.12	0.11	17.23	976.18
GO-1400	96.48	3.20	0.09	40.20	1250.67
GO-2000	98.55	0.64	0.04	205.30	2874.38
N-rGO*	70.55	17.34	10.33	5.42	7.97
N-rGO-1000*	81.33	10.69	6.73	10.14	14.10
N-rGO-1400*	96.39	1.03	2.37	124.80	47.44
N-rGO-2000*	99.36	0.27	0.18	490.70	644
rGO	69.83	25.86	0.22	3.60	370.31
N-rGO[#]	65.04	23.81	9.27	3.64	8.19

* refers to the N-rGO sheets with 10.3 wt% nitrogen or the graphene films made by such N-rGO sheets at differet temperatures.

[#] refers to the N-rGO sheets with 9.3 wt% nitrogen.

The number in the sample name indicates the annealing temperature (℃) .

Supplementary Table 2. XRD data and the structure features of N-rGO-, GO-, rGO-derived graphene films and HOPG. The number in the sample name indicates the annealing temperature (℃).

Sample	2θ position (°)	FWHM (°)	d-spacing (Å)	$P_{disorder}$ (%)	L_c § (nm)	L_c (nm)	L_a (μm)	AB-stacking (%)
N-rGO-2000*	26.039	0.624	3.4191	85.1	12.9	0.5	-	~0
N-rGO-2500*	26.458	0.230	3.3660	29.4	35.1	11.0	1.4	45.8
N-rGO-2800*	26.500	0.152	3.3607	19.5	53.1	28.8	12.5	96.9
N-rGO-3000*	26.539	0.102	3.3558	6.8	79.1	52.4	20.0	99.9
GO-2000	26.051	0.393	3.4176	84.0	20.5	1.3	-	~0
GO-2500	26.433	0.238	3.3691	34.3	33.9	3.5	-	22.0
GO-2800	26.450	0.219	3.3670	31.1	36.9	8.5	-	43.5
GO-3000	26.461	0.178	3.3656	28.7	45.3	11.7	2.7	77.8
rGO-3000	26.521	0.158	3.3581	13.4	51.1	16.5	3.2	92.3
N-rGO-3000#	26.502	0.131	3.3605	19.0	61.6	28.6	6.5	98.5
HOPG	26.541	0.150	3.3556	6.2	49.1	80.4	28.8	99.9

* refers to the graphene films made by N-rGO sheets with 10.3 wt% nitrogen.
# refers to the graphene films made by N-rGO sheets with 9.3 wt% nitrogen.
§ L_c was estimated by Scherrer equation for comparison.
L_c, L_a and the fraction of AB stacking were determined by ADF-STEM, SEM-ECC and Raman spectroscopy, respectively, as discussed in the manuscript.

Supplementary Table 3. Electrical and thermal conductivity evolution of GO, N-rGO and rGO films with annealing temperature.

**Temperature (℃)**	**N-rGO film***		**GO film**		**rGO film**	
	σ **(S cm^{-1})**	***K*** **(W m^{-1} K^{-1})**	***σ*** **(S cm^{-1})**	***K*** **(W m^{-1} K^{-1})**	***σ*** **(S cm^{-1})**	***K*** **(W m^{-1} K^{-1})**
2000	312.5	59.7	1325.7	451.2	363.1	64.1
2500	8119.3	1004.5	2447.0	740.1	3258	615.4
2800	13140.0	1433.2	3111.0	789.1	5959.7	934.8
3000	20000.0	1692.2	3254.2	883.2	9394.0	1006.4

* refers to the graphene films made by N-rGO sheets with 10.3 wt% nitrogen.

Supplementary Table 4. EMI shielding performances of various conductive materials.

**Sample**	**Thickness (μm)**	**Density ($g\ cm^{-3}$)**	**Frequency (GHz)**	**EMI SE (dB)**	***K*** **($W\ m^{-1}\ K^{-1}$)**	***σ*** **($S\ cm^{-1}$)**	**Ref.**
N-rGO-derived graphene film	10	2.22	8.2-12.4	90	1700	20000	This work
GO-derived graphene film	10	1.73	8.2-12.4	20	883.2	3254.2	This work
Graphene film (Gr/GO-1500°C)	50-70	1.0-1.6	8.2-12.4	75	700	2170	1
Graphene film (Gr-1000°C)	16	1.49	8.2-12.4	53.5	190	1340	2
Graphene film (rGO/CNT-2800°C)	15	1.45	2-18	57.6	1154	2740	3
Graphene film (rGO-2800°C)	13	0.91	2-18	48.9	510	1780	3
Graphene film (HI-rGO)	15	-	0.3-4	20	1390	243	4
Graphene film (GO-2000°C)	8.4	-	8.2-12.4	20	1100	1000	5
Graphene nanocomposite*	2700	-	8.2-12.4	55	4.13	-	6
MXene film ($Ti_3C_2T_x$)	11	2.39	8.2-12.4	68	55.8	4800	7

MXene film (Ti_3CNT_x-350°C)	10	-	8.2-12.4	75.1	-	2475	8
MXene composite film#	27	-	8.2-12.4	44.4	4.57	7.16	9
MXene composite film§	25	-	8.2-12.4	65	28.8	44.2	10

* refers to graphene nanocomposite containing carbonyl iron–nickel alloy powder and cyanate ester.
# refers to poly(vinyl alcohol)/$Ti_3C_2T_x$ multilayered films.
§ refers to $Ti_3C_2T_x$/montmorillonite laminated composite films.

References

1. Wang S, Sun X and Xu F *et al*. Strong yet tough graphene/graphene oxide hybrid films. *Carbon* 2021; **179**: 469-76.

2. Wei Q, Pei S and Qian X *et al*. Superhigh electromagnetic interference shielding of ultrathin aligned pristine graphene nanosheets film. *Adv Mater* 2020; **32**: 1907411.

3. Zhou E, Xi J and Guo Y *et al*. Synergistic effect of graphene and carbon nanotube for high-performance electromagnetic interference shielding films. *Carbon* 2018; **133**: 316-22.

4. Kumar P, Shahzad F and Yu S *et al*. Large-area reduced graphene oxide thin film with excellent thermal conductivity and electromagnetic interference shielding effectiveness. *Carbon* 2015; **94**: 494-500.

5. Shen B, Zhai W and Zheng W. Ultrathin flexible graphene film: an excellent thermal conducting material with efficient emi shielding. *Adv Funct Mater* 2014; **24**: 4542-8.

6. Ren F, Song D and Li Z *et al*. Synergistic effect of graphene nanosheets and carbonyl iron–nickel alloy hybrid filler on electromagnetic interference shielding and thermal conductivity of cyanate ester composites. *J Mater Chem C* 2018; **6**: 1476-86.

7. Shahzad F, Alhabeb M and Hatter CB *et al*. Electromagnetic interference shielding with 2D transition metal carbides (MXenes). *Science* 2016; **353**: 1137-40.

8. Iqbal A, Shahzad F and Hantanasirisakul K *et al*. Anomalous absorption of electromagnetic waves by 2D transition metal carbonitride

Ti_3CNT_x (MXene). *Science* 2020; **369**: 446-50.

9. Jin X, Wang J, Dai L *et al*. Flame-retardant poly(vinyl alcohol)/MXene multilayered films with outstanding electromagnetic interference shielding and thermal conductive performances. *Chem Eng J* 2020; **380**: 122475.

10. Li L, Cao YX and Liu XY *et al*. Multifunctional MXene-based fireproof electromagnetic shielding films with exceptional anisotropic heat dissipation capability and joule heating performance. *ACS Appl Mater Interfaces* 2020; **12**: 27350-60.
